# Prevalence and characteristics of tigecycline- and carbapenem-resistant *adeN*-truncated *Acinetobacter baumannii*: a genomic epidemiological analysis

**DOI:** 10.1128/aac.01843-24

**Published:** 2025-04-23

**Authors:** Ying Zhang, Beibei Zhou, Jingchun Kong, Panjie Hu, Haifeng Liu, Deyi Zhao, Jianzhong Ye, Qingxia Fu, Tieli Zhou, Changrui Qian

**Affiliations:** 1 Department of Clinical Laboratory, Key Laboratory of Clinical Laboratory Diagnosis and Translational Research of Zhejiang Province, The First Affiliated Hospital of Wenzhou Medical University 89657, Wenzhou, Zhejiang, China; 2 School of Laboratory Medicine and Life Science, Wenzhou Medical University 214181, Wenzhou, Zhejiang, China; Johns Hopkins University School of Medicine, Baltimore, Maryland, USA

**Keywords:** *Acinetobacter baumannii*, *adeN*, prevalence, antimicrobial resistance, genomic analysis

## Abstract

*adeN*-truncated *Acinetobacter baumannii* (ATAB) isolates are associated with elevated tigecycline resistance and enhanced virulence, yet its epidemic dynamics and genomic features remain poorly understood. This study aimed to investigate the epidemiology of ATAB isolates, identify infection risk factors, and assess their impact on patient prognosis. The prevalence of ATAB isolates in a tertiary care teaching hospital (Wenzhou, China) from January 2018 to December 2022 was determined via polymerase chain reaction (PCR) screening. Whole-genome sequencing and genomic analysis were conducted to explore the epidemiology and genomic characteristics of 254 ATAB isolates. Clinical data analysis was performed to identify risk factors for ATAB infection and its correlation with patient prognosis. The results of local sample analysis showed that *adeN* truncation was identified in 26.5% (486/1834) of the patient isolates, with the monthly prevalence peaking at 64.9% (24/37). The capsular types of ATAB isolates were primarily KL2, whereas *adeN*-complete isolates exhibited a greater capsular diversity. ATAB could evolve within the hospital and lead to multiple nosocomial clonal transmissions. Most ATAB isolates exhibited co-resistance to carbapenems and tigecycline. ICU admission and use of immunosuppressants were significant risk factors for ATAB isolate infection. Patients infected with ATAB isolates had significantly higher 28-day all-cause mortality (32.8%, 20/61) compared to those infected with *adeN*-complete isolates (11.5%, 7/61, *P* < 0.01). Bioinformatics analysis of the 18,946 completed and draft *A. baumannii* genome assemblies from the GenBank database showed that ATAB isolates were widely prevalent worldwide. This study highlights the importance of early identification and monitoring of ATAB isolates as a critical marker for hospital infection control.

## INTRODUCTION

*Acinetobacter baumannii* is a major nosocomial pathogen, frequently causing life-threatening infections such as pneumonia, meningitis, and bloodstream infections (1). Its ability to develop resistance to multiple antimicrobial agents, particularly carbapenems, has made treatment options increasingly limited (2, 3). Tigecycline is often regarded as one of the last lines of defense against carbapenem-resistant *A. baumannii* (CRAB). However, the emergence of tigecycline-resistant isolates has been widely reported, primarily driven by the overexpression of RND efflux pumps, such as AdeABC and AdeIJK, which can reduce tigecycline susceptibility to varying degrees (4–7).

AdeN, a TetR family transcriptional regulator, critically regulates the expression of AdeIJK, the most conserved RND efflux pump (8). In 2017, Rajagopalan *et al*. first reported an IS*Aba1* insertion in the *adeN* gene of clinical *A. baumannii* isolates, demonstrating that this genetic alteration increases the lethality of *A. baumannii* ATCC 17978 against A549 lung cells (9). IS*Aba1*-mediated truncation of *adeN* has also been identified in multiple studies as a key resistance-associated genetic alteration, linked to tigecycline resistance (5–7). Despite these insights, the epidemiological characteristics of *adeN*-truncated *A. baumannii* (ATAB) isolates are not yet well understood. Moreover, the prognostic outcomes of patients infected with ATAB isolates, compared to those with *adeN*-complete isolates, remain to be further elucidated. To address these gaps, this study retrospectively screened *A. baumannii* isolates collected over a 5-year period from a large tertiary care hospital for *adeN* truncations. Through whole-genome sequencing and genomic database mining, we systematically investigated the epidemiological characteristics of ATAB isolates and analyzed potential infection risk factors and clinical outcomes using comprehensive clinical data.

## RESULTS

### ATAB isolates are prevalent in hospital patients

**Fig 1:**
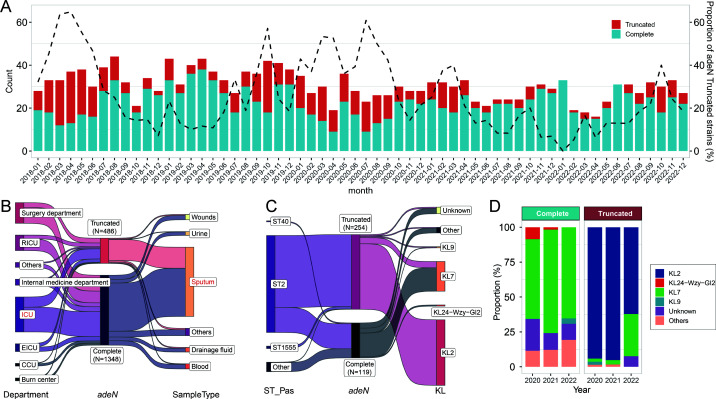
Epidemiological characteristics and distribution of *adeN*-truncated and *adeN*-complete *A. baumannii* isolates in the hospital. (**A**) Monthly distribution of *adeN*-truncated and *adeN*-complete isolates over time. (**B**) Distribution of *adeN*-truncated and *adeN*-complete isolates in different hospital departments and sample types. (**C**) Molecular typing of isolates with different *adeN* statuses. (**D**) Temporal distribution of KL types of *adeN*-truncated and *adeN*-complete isolates.

A total of 1,834 *A*. *baumannii* isolates from patients were collected between January 2018 and December 2022 from a large tertiary care hospital to investigate the prevalence of ATAB isolates. These isolates were derived from various clinical specimens collected from patients, including sputum (72.8%, 1,336/1,834), wounds (5.5%, 101/1,834), and blood (5.3%, 97/1,834) (Fig. S1). The polymerase chain reaction (PCR) screening result showed that 26.5% (486/1,834) of *A. baumannii* isolates harbored truncated *adeN*. The proportion of ATAB isolates showed considerable fluctuations over the years, peaking at 64.9% (24/37) in April 2018 (Fig. 1A). The ATAB isolates were primarily derived from sputum specimens of various ICU patients (Fig. 1B).

WGS was performed on all ATAB isolates (*N* = 254) from 2020 to 2022, alongside randomly selected *adeN*-complete strains (*N* = 119) from the same period. The metadata for these strains have been provided as the supplementary table (Table S1). These ATAB isolates predominantly belonged to the IC2/ST2 clone (96.5%, 245/254), and the main capsule type was KL2 (87.8%, 223/254) (Fig. 1C). In contrast, *adeN*-complete strains exhibited greater diversity in MLST and capsule types, with ST2-KL7 being most common (66.4%, 79/119) and no KL2 clones identified. By analyzing the capsule-type distribution over time, we found that *adeN*-complete KL7 strains maintained a consistent prevalence, whereas *adeN*-truncated KL7 strains expanded significantly in 2022 (Fig. 1D).

### Frequent clonal dissemination of ATAB isolates in nosocomial settings

**Fig 2:**
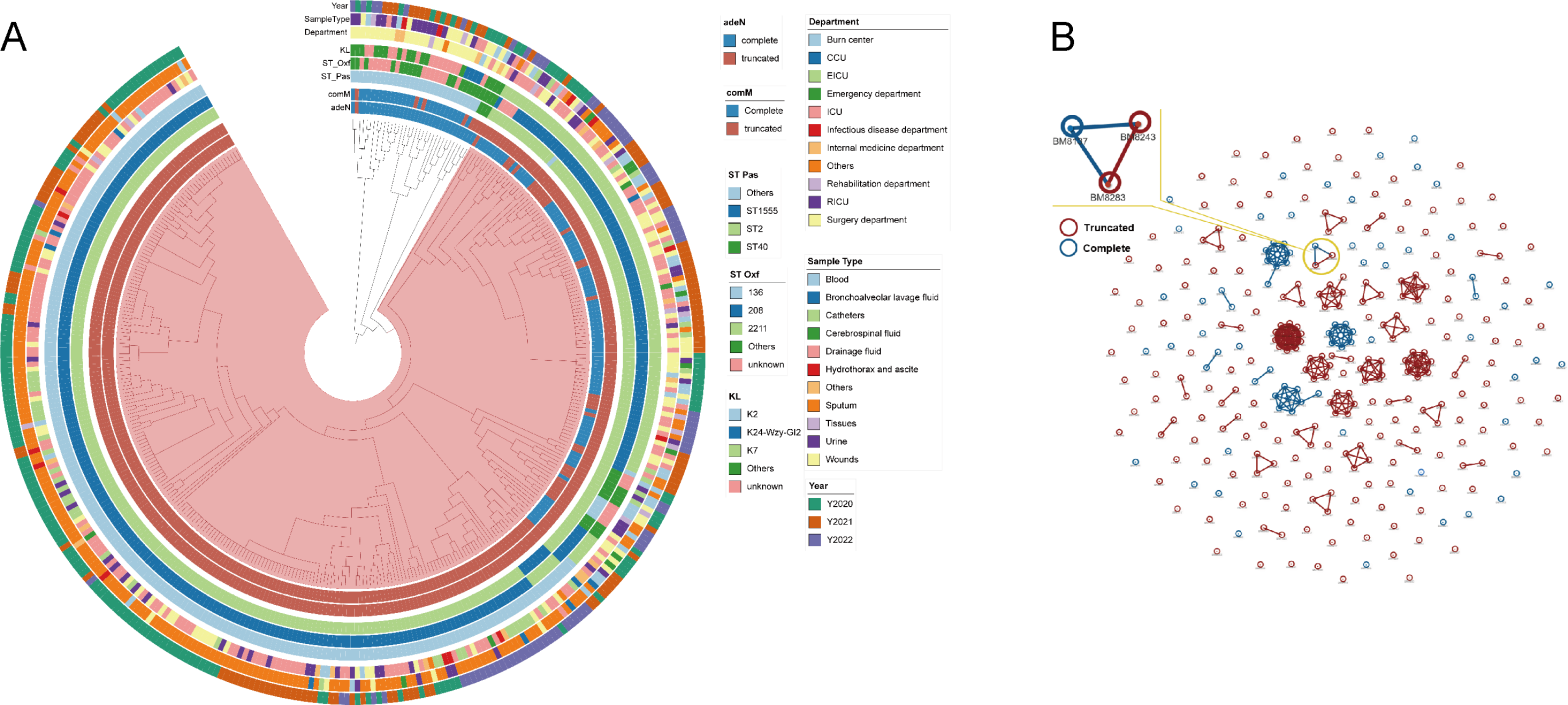
Phylogenetic analysis of 373 sequenced *A. baumannii* isolates (**A**) Maximum-likelihood cgSNP-based phylogenetic tree. The rings from inside to outside represent the isolate’s *adeN* status, *comM* status, sequence type of the Pasteur scheme, sequence type of the Oxford scheme, K type, department, sample type, and year, respectively. (**B**) Genetic clusters based on cgSNPs with an SNP distance of 0. Red circles represent ATAB isolates, blue circles represent *adeN*-complete isolates, and the connecting line represents that the cgSNP distance between isolates is 0.

To investigate the relationships between ATAB and *adeN*-complete isolates, we constructed a cgSNP-based phylogenetic tree (Fig. 2A). We found that the ST2^Pas^/ST208^Oxf^-KL2 lineage formed the largest ATAB branch, widely distributed across multiple departments. The *adeN*-complete isolates were mainly composed of the ST2^Pas^/ST208^Oxf^-KL7 lineage. We clustered all ST2^Pas^/ST208^Oxf^-KL2 and ST2^Pas^/ST208^Oxf^-KL7 isolates using a cgSNP distance of 0 to search for potential clonal transmission (Fig. 2B). Interestingly, we found that ATAB isolates formed 17 clusters, involving up to 35.2% (83/236) of the isolates, while *adeN*-complete isolates formed only three genetic clusters, involving 30.7% (23/75) of the isolates. This indicated ATAB isolates have driven significant clonal transmission in the hospital. Notably, within one cluster, *adeN*-complete isolates BM8137 evolved to ATAB BM8243 and BM8283, suggesting *adeN* truncation occurs in the nosocomial environment.

### Global emergence and prevalence of ATAB isolates

**Fig 3:**
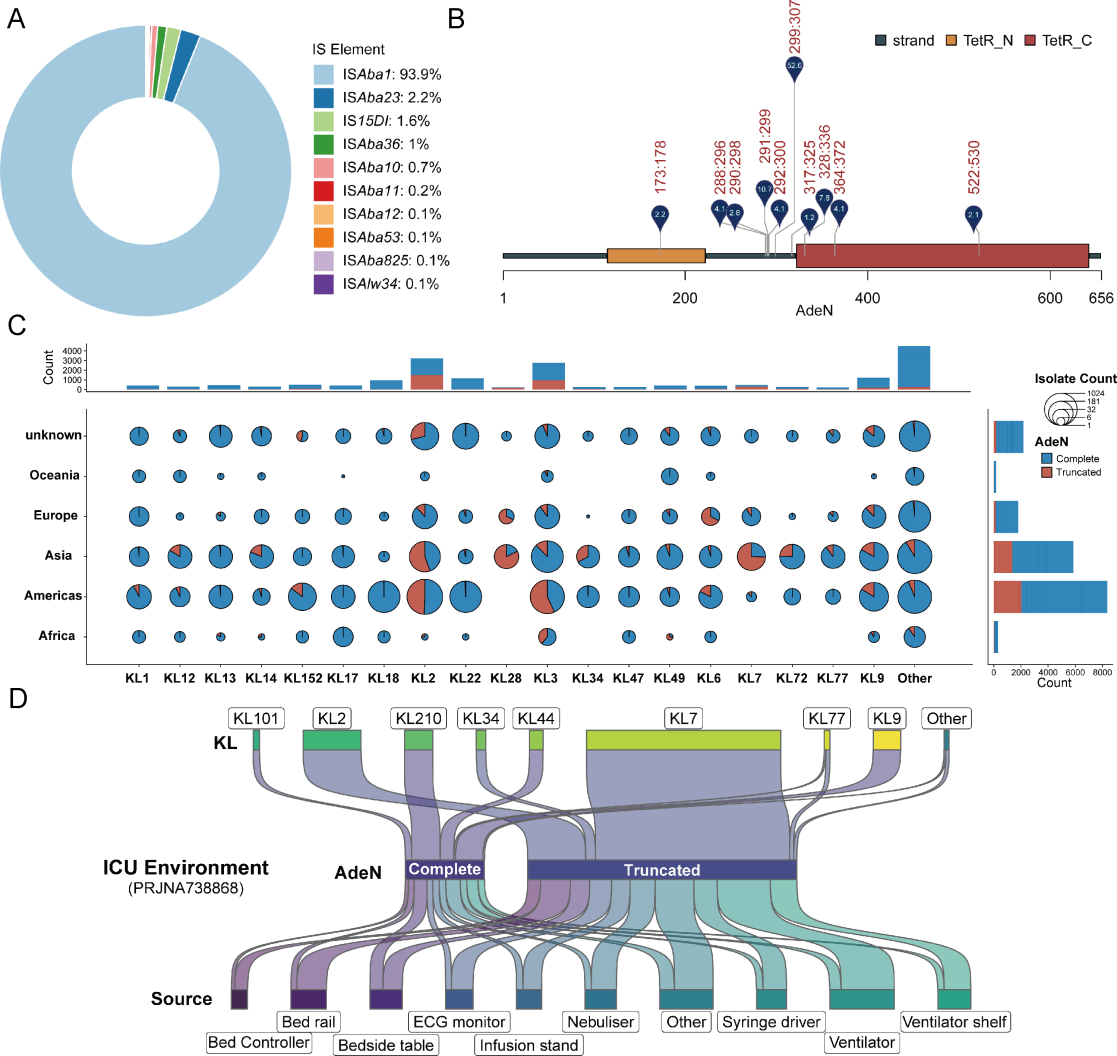
Global distribution and genomic characteristics of *adeN*-truncated and *adeN*-complete *A. baumannii* isolates. (**A**) Prevalence of different IS elements associated with truncation of the *adeN* gene. (**B**) Schematic of the *adeN* gene with truncation sites caused by IS elements. Only sites with a frequency higher than 2% are shown. The numbers next to the markers indicate the proportion of strains with truncations at each site. (**C**) Distribution of KL types among *adeN*-truncated and *adeN*-complete isolates across different continents. Bubble sizes represent the number of strains, with pie chart segments indicating the proportions of truncated versus complete *adeN* isolates. (**D**) Source distribution of *adeN*-truncated and *adeN*-complete isolates in the ICU environment (PRJNA738868).

To investigate the global prevalence of ATAB isolates, we analyzed 18,946 high-quality *A. baumannii* genomes from the GenBank database. Our results revealed that these ATAB isolates were widespread across numerous regions (Fig. S2). IS*Aba1* was the dominant IS element causing truncation (93.9%), followed by IS*Aba23* (2.2%) (Fig. 3A). Notably, 52.6% of insertions occurred between positions 299 and 307 of the *adeN* gene (Fig. 3B). Consistent with local observations, the IC2 clone was the predominant genotype of ATAB isolates across continents (Fig. S3). Among the five major IC2-associated capsule types (KL2, KL3, KL7, KL9, and KL22), KL22 primarily comprised *adeN*-complete strains, while the others showed varying degrees of *adeN* truncation (Fig. S4). Notably, *adeN*-truncated KL2 strains were widespread globally, KL3 strains were predominant in the Americas, and KL7 strains were mainly found in Asia (Fig. 3C). Interestingly, ATAB strains were first identified in Europe in 2003 and have since spread widely across America and Asia (Fig. S5). These findings underscore the global prevalence of ATAB isolates and their strong association with specific genetic traits.

Importantly, we expanded our analysis to include a recently published large-scale genomic data set from another hospital in Zhejiang Province. This data set comprised 432 strains isolated from various medical device surfaces in the ICU ward between August and October 2019 (10). Surprisingly, we found that ATAB isolates were highly prevalent (73%, 334/432) across all types of surfaces in the ICU (Fig. 3D). The capsule type of these ATAB isolates was predominantly KL7 (72.2%, 241/334), followed by KL2 (21.6%, 72/334). This finding highlights the widespread presence of ATAB isolates in ICU environments.

### High correlation of ATAB isolates with carbapenem resistance

**Fig 4:**
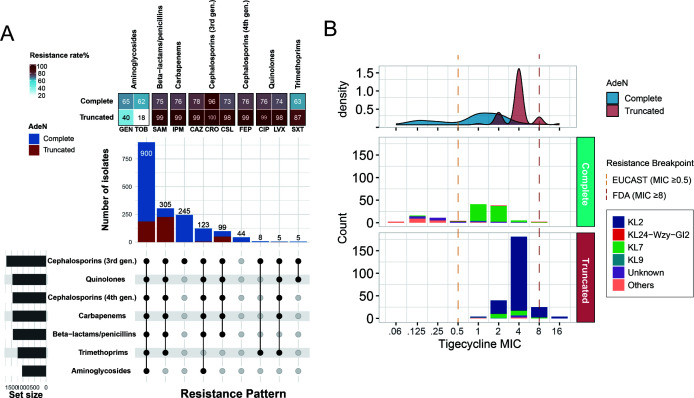
Antimicrobial susceptibility phenotypes of *adeN*-truncated and *adeN*-complete *A. baumannii* isolates. (**A**) Resistance rates and pattern of ATAB and *adeN*-complete isolates across different antimicrobials. The heatmap at the top indicates the resistance percentage, while the bar plot in the middle shows the number of isolates resistant to each antibiotic. The bottom section presents an UpSet plot of resistance patterns across different antibiotic classes. (**B**) Distribution of tigecycline MICs for *adeN*-truncated and *adeN*-complete isolates. The density plot at the top illustrates the distribution of MIC values, while the bar plot below shows the count of isolates. The vertical lines represent the tigecycline resistance breakpoints according to EUCAST and FDA guidelines.

We compared the drug resistance profiles of 1,765 isolates with complete susceptibility data for 11 antibiotics (Fig. 4A). ATAB isolates showed significantly higher resistance to cephalosporins, carbapenems, and quinolones, with rates approaching or exceeding 99% (*P* < 0.001). However, ATAB isolates had lower resistance to aminoglycosides (*P* < 0.001). Further analysis of resistance patterns revealed that 65% of ATAB isolates exhibited multidrug-resistant phenotypes, while 35% were extensively drug-resistant. Carbapenem resistance in *A. baumannii* is primarily mediated by carbapenem-hydrolyzing class D β-lactamases, such as *bla*_OXA-23_, which are typically located within AbaR-type resistance islands (AbaRs) (11, 12). Previous studies have demonstrated that AbaRs are predominantly inserted into the *comM* gene, which encodes a helicase involved in DNA recombination during natural transformation (13, 14). Interestingly, we observed that most of the ATAB isolates in both local and global data sets also exhibited truncations in the *comM* gene (Fig. 2A; Fig. S6). This suggested that *adeN* truncation predominantly occurs in strains where the AbaR island has integrated into *comM*. Overall, these findings highlight a strong association between ATAB and antimicrobial resistance, emphasizing the urgent need for targeted interventions in clinical settings.

### Genetic background affects tigecycline resistance of ATAB isolates

Given the association between *adeN* truncation and reduced tigecycline susceptibility, we further analyzed tigecycline MICs in 373 sequenced isolates. The results showed that tigecycline MICs for ATAB isolates were generally high, ranging from 1 to 16 mg/L, with an MIC_50_ value of 4 mg/L (Fig. 4B). Based on EUCAST breakpoints, all ATAB isolates were tigecycline-resistant, whereas FDA standards classified only 11.4% (29/254) ATAB isolates as resistant. In contrast, tigecycline MICs for *adeN*-complete isolates were more widely distributed, ranging from 0.06 to 8 mg/L, and were correlated with the capsular types of isolates. Notably, *adeN*-complete KL7 strains exhibited relatively high MIC values, with an MIC_50_ of 2 mg/L, approaching that of *adeN*-truncated KL7 strains (MIC_50_ of 4 mg/L). Other capsular-type strains displayed lower tigecycline MIC values, with an MIC_50_ of 0.125 mg/L. These results update our understanding of the association between *adeN* truncation and tigecycline resistance phenotype.

### Infection of ATAB isolates is associated with worse patient outcomes

**Fig 5:**
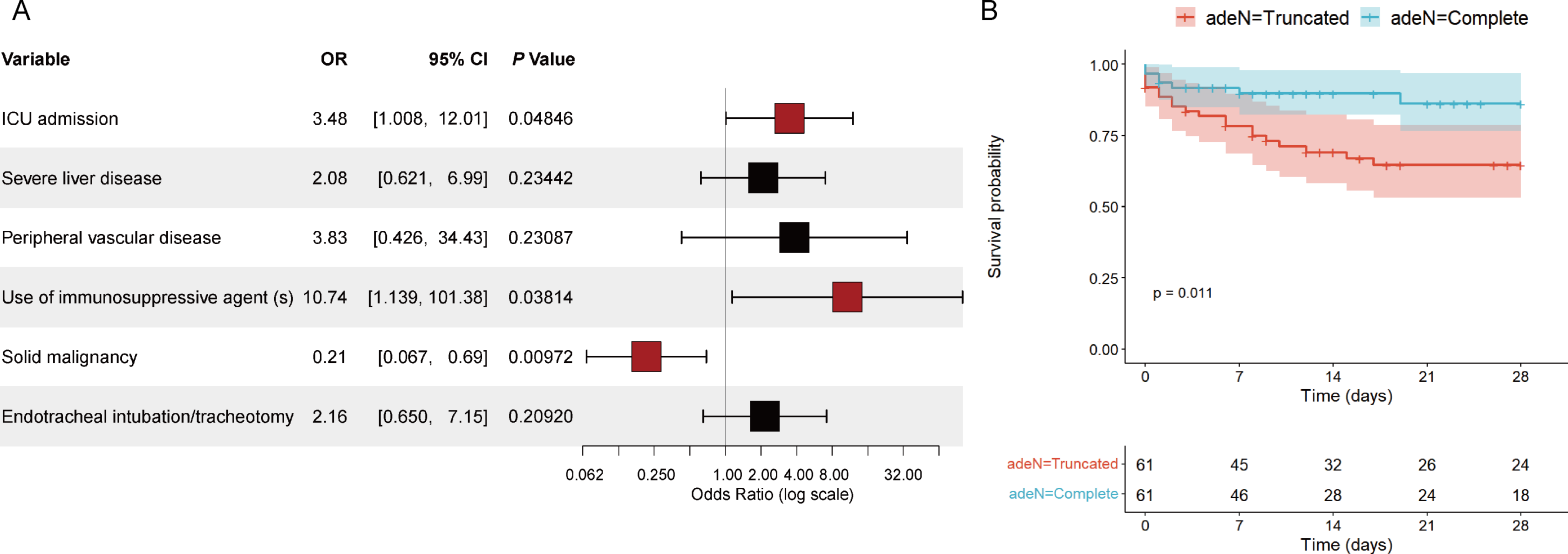
Clinical data analysis of patients infected with *adeN*-truncated and *adeN*-complete isolates. (**A**) Forest plot of risk factors associated with infection by *adeN*-truncated *A. baumannii* isolates. Odds ratios (OR) with 95% confidence intervals (CI) are displayed on a logarithmic scale. The red squares indicate variables with significant *P*-values (*P* < 0.05). (**B**) Kaplan–Meier survival curves in patients with *A. baumannii* isolate infection.

To evaluate the specific impact of infection with ATAB isolates on patient clinical outcomes, this study included 61 patients from whom ATAB isolates were isolated from sterile body fluids. As a control, 61 patients with *adeN*-complete strains isolated were randomly selected through case-matching. There were no significant differences between the two groups in terms of gender, age distribution, and Charlson Comorbidity Index (Table S2). Notably, we observed that patients in the ATAB group had a significantly longer pre-infection hospital stay (median 42 days vs 27 days, *P* < 0.05) and higher ICU admission rate (75.4% vs 39.3%, *P* < 0.001). Additionally, the ATAB group was more likely to suffer from severe liver disease (29.5% vs 11.5%, *P* < 0.05), peripheral vascular disease (16.4% vs 1.6%, *P* < 0.01), immunosuppressant use (16.4% vs 1.6%, *P* < 0.01), and receiving tracheal intubation or tracheostomy (73.7% vs 44.3%, *P* < 0.001). In contrast, the incidence of solid malignancies was higher in the *adeN*-complete group (31.1% vs 11.5%, *P*<0.01) (Table S2). Further logistic regression analysis of these significant factors identified ICU admission and immunosuppressant use as independent risk factors for infection with ATAB isolates (Fig. 5A). Patients infected with ATAB exhibited higher 14-day (29.5%, 18/61) and 28-day (32.8%, 20/61) mortality rates compared to those infected with *adeN*-complete *A. baumannii* (*P* < 0.01) (Table S2). Furthermore, the Kaplan–Meier curves with the log-rank test revealed a significant difference in 28-day survival between the two groups (*P*  <  0.05) (Fig. 5B).

### ATAB has higher virulence and reduced biofilm formation ability

**Fig 6:**
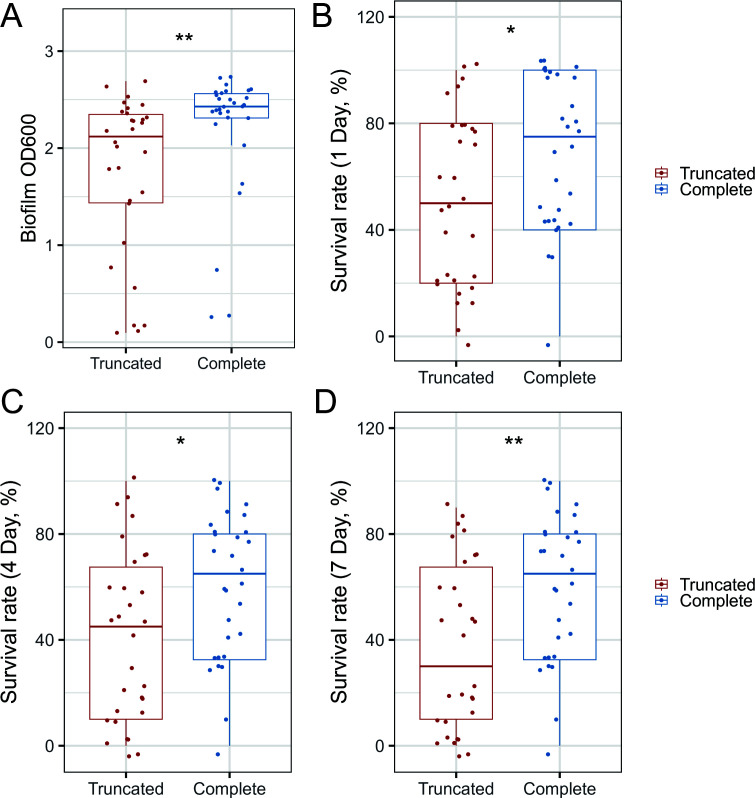
Comparison of virulence traits between the *adeN*-truncated and *adeN*-complete isolates. (**A**) Biofilm formation. (**B–D**) Survival plot of *Galleria mellonella* larvae infected with the *adeN*-truncated group compared to the *adeN*-complete group. (**B**) Observed for 1 day. (**C**) Observed for 4 days. (**D**) Observed for 7 days.

To assess the virulence characteristics of patient-isolated *adeN*-truncated strains, we randomly selected 30 *adeN*-truncated and 30 *adeN*-complete *A. baumannii* clinical isolates for subsequent experiments. Biofilm formation was compared between the two groups using crystal violet staining, revealing that *adeN*-truncated *A. baumannii* strains exhibited significantly weaker biofilm formation ability compared to the *adeN*-complete strains (Fig. 6A). Furthermore, we assessed the *in vivo* pathogenicity of the two group isolates using the *G. mellonella* larvae infection model. The results demonstrated that the 1-day, 4-day, and 7-day survival rates of *G. mellonella* larvae infected with the *adeN*-truncated strains were lower compared to those infected with the *adeN*-complete strains (Fig. 6B through D).

## DISCUSSION

This study comprehensively explored the genomic epidemiological characteristics of *adeN*-truncated *A. baumannii* at both local and global levels. Our results reveal a widespread prevalence of *adeN*-truncated *A. baumannii* in hospital settings. This strain often exhibits more severe antimicrobial resistance characteristics and is closely associated with poor prognoses in infected patients. Our findings emphasize the potential value of *adeN* truncation in guiding clinical treatment and prognosis assessment for infected patients. Furthermore, our study highlights a close association between *adeN*-truncated *A. baumannii* and a specific global epidemic lineage, which enriches our understanding of the epidemic dynamics of *adeN*-truncated *A. baumannii*.

The IC2 lineage of *A. baumannii* is a globally successful, carbapenem-resistant clonal group that has caused numerous outbreaks of hospital-acquired infections worldwide (15–17). Our study demonstrated that *adeN* truncation predominantly occurs in the IC2 clonal group, with KL2 and KL3 being the primary capsular types in the Americas and KL2 and KL7 in Asia. Notably, we also observed the expansion of KL7-type ATAB strains, which is consistent with our previous studies (18). Several studies have shown that KL7 has a higher virulence than other capsule types and has caused outbreaks in multiple hospitals, suggesting that it has a good fitness advantage in hospitals (19, 20). Although the driving factors behind ATAB isolate dissemination remain unclear, previous studies have shown that disinfectants significantly increase the frequency of *adeN* inactivation in *A. baumannii* (21, 22). Taking these factors into consideration, we speculate that the differences in genotypes (such as capsule types) of ATAB strains may be related to the frequent exposure of local hospital epidemic lineages to disinfectant-induced selection pressure. Based on the phylogenetic analysis, we also observed that ATAB evolved within the hospital and led to multiple clonal transmissions, which may also be the driving factor for its expansion. Notably, *adeN* truncation may impose an adaptive burden on *A. baumannii* due to overexpression of the AdeIJK efflux pump, potentially limiting the competitive advantage of ATAB isolates in environments without selective pressures (23, 24). These multifaceted factors likely contribute to the periodic prevalence of ATAB isolates in hospital settings.

We also observed an intriguing association between ATAB isolates and specific resistance patterns, particularly resistance to cephalosporins, carbapenems, fluoroquinolones, and tigecycline. Although AdeIJK efflux pump substrates include β-lactams, fluoroquinolones, and tetracyclines, its contribution to resistance appears limited (21, 23). We hypothesize that genomic differences in resistance determinants within ATAB lineages also play a significant role. Notably, for tigecycline resistance, we found that the *adeN*-complete KL7 lineage itself has a high MIC value for tigecycline (MIC_50_ = 1). Studies have demonstrated that high-level tigecycline resistance in *A. baumannii* is frequently associated with the overexpression of the AdeIJK efflux pump or the presence of plasmid-borne tigecycline-inactivating enzyme TetX variants (5, 25). Furthermore, specific mutations within chromosomal genes, such as *trm* and *plsC*, may contribute to diminished tigecycline susceptibility (26, 27). We speculate that the accumulation of tigecycline resistance-associated mutations in the KL7-type *A. baumannii* isolates resulted in reduced sensitivity to tigecycline. Therefore, when *adeN* truncation occurs, it leads to clinical-level resistance in strains. For other lineages, however, the occurrence of *adeN* truncation may not be sufficient to induce clinical resistance to tigecycline. This underscores the importance of considering the genetic background of the strains when interpreting the relationship between *adeN* truncation and clinical tigecycline resistance.

*A. baumannii* primarily infects immunocompromised hospitalized patients, with infection-related mortality rates ranging from 34% to 44.5% (28). Previous studies have shown that the mortality rate of patients infected with *A. baumannii* is closely related to the severity of infection, antimicrobial resistance, timeliness of treatment, and the lineage of the infecting strain (29, 30). In the present study, a statistically significant elevation in mortality risk was observed among patients infected with ATAB isolates. Consistent with previous reports, we demonstrated enhanced lethality of ATAB strains in the *Galleria mellonella* larval infection model (9). Considering the multifaceted characteristics of ATAB strains, we hypothesize that the increased mortality risk may be attributed to a confluence of factors, including (i) virulence enhancement due to *adeN* gene truncation; (ii) complex antimicrobial resistance profiles, potentially contributing to clinical treatment failure; and (iii) intrinsic pathogenic properties associated with the prevalent ATAB lineage. Collectively, these findings underscore the potential clinical utility of *adeN* truncation as an early indicator for guiding therapeutic interventions and prognostic assessments in *A. baumannii* infections.

This study has several limitations. First, as a single-center analysis, the generalizability of conclusions regarding the genetic characteristics and prevalence of ATAB isolates may be limited. Second, the small number of isolates from sterile sites constrained our ability to fully assess the potential impact of factors beyond the *adeN* status on patient outcomes. Furthermore, the inherent challenge of differentiating between true infection and colonization represents a notable limitation. While our study included only patients with isolates from sterile body fluids, the diagnosis of pneumonia was often based on clinical criteria and microbiological findings from nonsterile sites such as sputum or bronchoalveolar lavage. Furthermore, selection bias and the number of *adeN*-complete isolates used for WGS may limit the validity of some comparisons.

In conclusion, this study highlights the widespread presence of ATAB isolates as a potential high-risk clone. Given its association with resistance and virulence, early identification and monitoring of ATAB as a marker for hospital infection control are crucial for developing effective management and treatment strategies. Furthermore, the role of environmental disinfectants in driving the emergence and spread of ATAB warrants careful consideration.

## MATERIALS AND METHODS

### Collection and screening of *adeN*-truncated isolates

This retrospective study was conducted at the First Affiliated Hospital of Wenzhou Medical University, a large tertiary care teaching hospital in Zhejiang, China. *A. baumannii* isolates collected from January 2018 to December 2022 were used as experimental strains. The isolates were identified using matrix-assisted laser desorption/ionization time-of-flight mass spectrometry (MALDI-TOF/MS; bioMérieux, Lyon, France). These isolates were preserved in 30% glycerol broth vials and stored at −80°C for future use. The integrity of the *adeN* gene in *A. baumannii* isolates was assessed using PCR. An *adeN* truncation was indicated if the PCR product band size after gel electrophoresis was 800 bp larger than that of *A. baumannii* ATCC 17978 (primer sequences are detailed in Table S3).

### Antimicrobial susceptibility testing

The susceptibility of strains to clinically used antibiotics, including amikacin, ceftazidime, ciprofloxacin, ceftriaxone, cefepime, gentamicin, imipenem, levofloxacin, ampicillin/sulbactam, cotrimoxazole and tobramycin, was determined using the automated broth microdilution method with VITEK2 (bioMérieux, Marcy-l'Étoile, France). Tigecycline susceptibility was determined using the manual broth microdilution method. Results for all other antimicrobial agents were interpreted according to the latest CLSI guidelines, while tigecycline susceptibility was interpreted based on EUCAST and FDA guidelines.

### Whole-genome sequencing, genome assembly, and annotation

Genomic DNA from 373 *A. baumannii* isolates, including both *adeN*-truncated and *adeN*-complete strains, was extracted using the AxyPrep Bacterial Genomic DNA Miniprep Kit (Axygen Scientific, USA). The DNA library was prepared using the NEBNext Ultra II DNA library preparation kit and subsequently sequenced by the Illumina NovaSeq. The quality control of raw sequence reads was performed using FastQC (https://github.com/s-andrews/FastQC) and trimmed using fastp with default parameters (31). The trimmed reads for each strain were assembled using SPAdes, and draft assemblies were annotated with Prokka (32, 33). The multi-locus sequence type (MLST) analysis of *A. baumannii* was performed using pymlst (34). The capsular (K-locus) and O-antigen (O-type) genotypes were determined using Kaptive (35). The antimicrobial resistance genes (ARGs) on *A. baumannii* were identified using AMRFinderPlus (36). The Insertion Sequence (IS) elements inserted into the *adeN* gene were identified using ISfinder (37).

### Phylogenetic analysis

The core genome alignment of genomes was identified using Snippy (https://github.com/tseemann/snippy), with the complete genome of BM2333, an IC2/ST2 strain previously isolated in our hospital, serving as the reference for comparison. The recombination site of generated alignment was removed using Gubbins (38). SNP distances were calculated from the Gubbins-filtered polymorphic sites file using SNP-dists (https://github.com/tseemann/snp-dists). Clonal transmission clusters with a core-genome SNP (cgSNP) distance of 0 were extracted using the R library iGRAPH (39).

### Analysis of *A. baumannii* assemblies from GenBank

A total of 18,946 completed and draft *A. baumannii* genome assemblies were downloaded from GenBank as of Jun 2023. The AST phenotype data of *A. baumannii* were retrieved from NCBI Pathogen Detection (www.ncbi.nlm.nih.gov/pathogens/). The completeness of the *adeN* gene in the *A. baumannii* genomes was checked using a self-written Python script (https://github.com/qcrcherry/comparative_genomic_analysis).

### Clinical data collection and definitions

To minimize errors caused by colonization, the study focused on adult patients (aged 18 and above) with the ATAB strain isolated from sterile body fluids (e.g., blood, pleural or peritoneal fluid, and cerebrospinal fluid). These patients formed the study group, while patients with *adeN*-complete strains from the same sample types were randomly selected as controls. Outpatient and duplicate cases were excluded. The Charlson Comorbidity Index was used to adjust for underlying diseases, and all-cause in-hospital mortality was recorded. The infection date was defined as the first isolation of *A. baumannii* from sterile body fluids. Infection sites were classified according to CDC guidelines, and laboratory results from the day of strain isolation were recorded. The primary outcome was the all-cause mortality rate within 28 days after *A. baumannii* infection.

### Biofilm formation assay

The biofilm-forming ability of *A. baumannii* was assessed using the crystal violet staining method. Bacterial suspensions were first adjusted to a concentration of 1.5 × 10^8^ CFU/mL. The suspensions were then diluted 100-fold with the LB medium and added to wells of a polystyrene 96-well plate (Corning, USA). After 24 hours of static incubation, the wells were stained with crystal violet (Solarbio, China), and excess stain was removed by washing with phosphate-buffered saline (PBS), followed by washing with absolute ethanol. The absorbance at 600 nm was measured to determine the average optical density of the triplicates. The experiment was repeated three times to obtain the mean value.

### *In vivo G. mellonella* survival rate analysis

The virulence of *A. baumannii* was assessed using an *in vivo G. mellonella* infection model (40). *G. mellonella* larvae weighing between 250 and 300 mg were used for the infection experiment. PBS served as the negative control. Overnight bacterial cultures were washed with PBS and resuspended to an OD_600_ of 0.5. A 10 µL aliquot of a tenfold dilution of each bacterial strain was injected into the left forelimb of each larva, with 10 larvae per group. The larvae were maintained at 37°C, and their survival was monitored continuously for 7 days post-injection. Survival rates were recorded at the corresponding time points.

### Statistical analysis and visualization

Data processing and statistical analyses were conducted using R v4.2. Categorical variables were reported as numbers (percentages) and compared using χ or Fisher’s exact tests, while continuous variables were presented as mean ± standard deviation or median (interquartile range) and analyzed using *t*-tests or Mann–Whitney U tests. A significance level of *P* < 0.05 was applied. A world map was built using the R package “rworldmap.” The Sankey diagram was produced by “ggsankey.” Survival analysis was performed by “survival.” The Kaplan–Meier curve was generated using “survminer.” The UpSet plot was generated using “ComplexUpset” v1.4.0. Univariate and multivariate logistic regression analyses were performed to analyze the risk factors for infection with the ATAB isolates, and the results were visualized using the “forestplot.”

## Data Availability

The genome data of sequenced isolates have been submitted to the NCBI under BioProject Accession number PRJNA1190325.

## SUPPLEMENTAL MATERIAL

Supplemental material aac.01843-24-s0001.docx Fig. S1 to S6; Tables S1 to S3.

ASM does not own the copyrights to Supplemental Material that may be linked to, or accessed through, an article. The authors have granted ASM a non-exclusive, world-wide license to publish the Supplemental Material files. Please contact the corresponding author directly for reuse.
