# Supplementary material for: Prevalence and characteristics of tigecycline- and carbapenem-resistant adeN-truncated Acinetobacter baumannii: a genomic epidemiological analysis
Source: Antimicrob Agents Chemother. 2025 Apr 23;69(6):e01843-24. doi: 10.1128/aac.01843-24 (PMC12135514; doi:10.1128/aac.01843-24)
Supplement: Supplemental material — Fig. S1 to S6; Tables S1 to S3. [file aac.01843-24-s0001.docx]

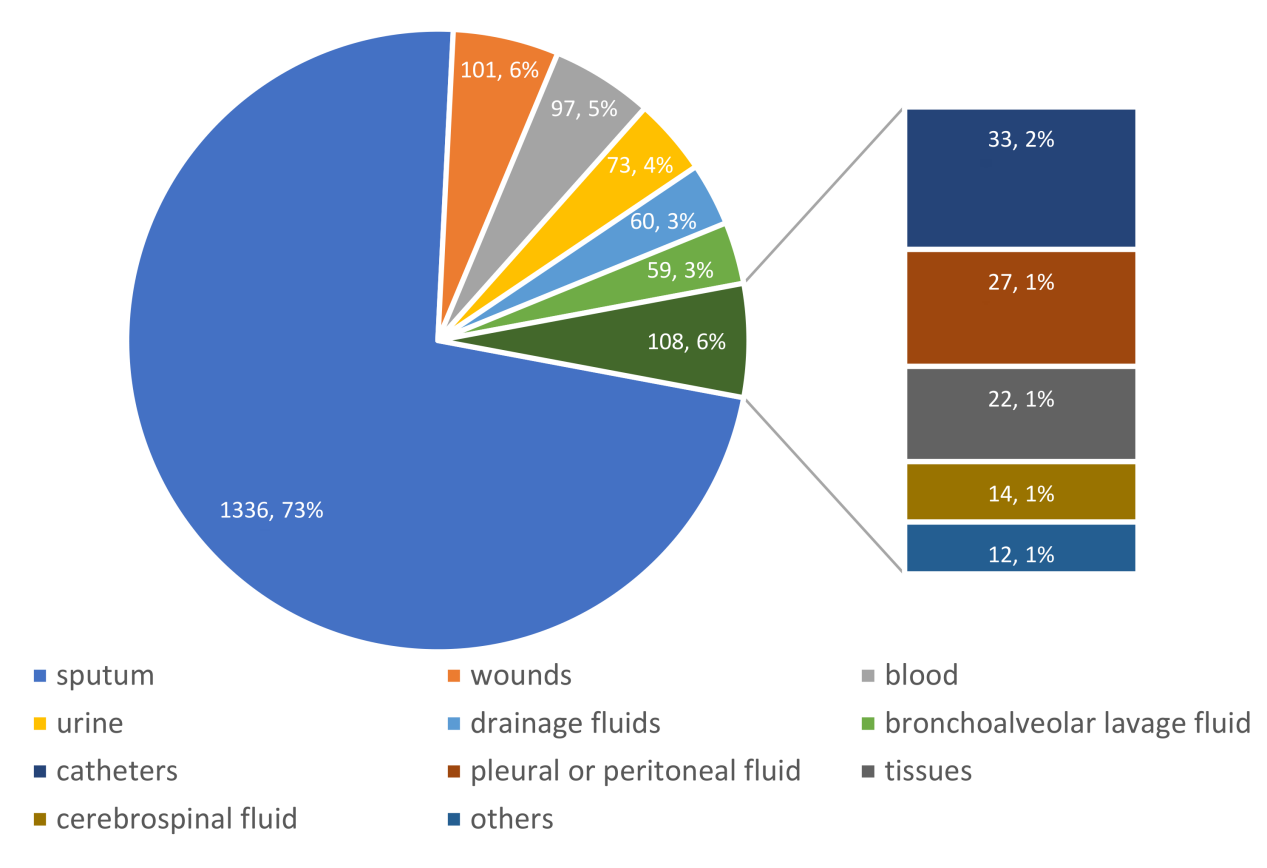


**Figure S1. Distribution of specimen types for *A. baumanni*i isolates from clinical patients.**

**
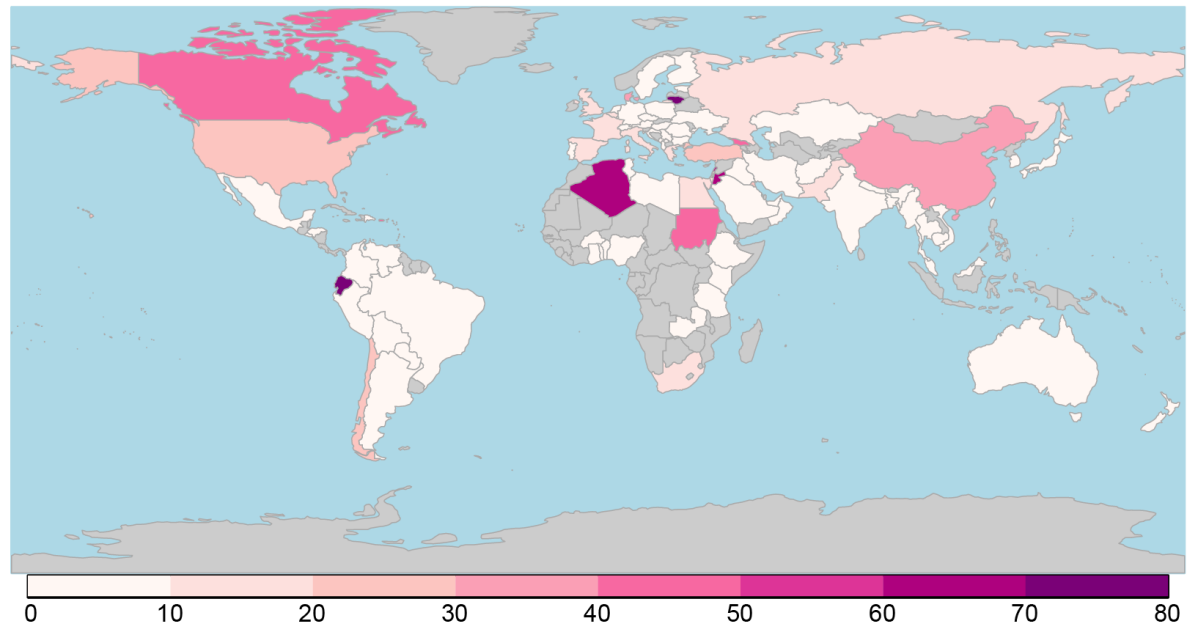
**

**Figure S2 Global geographic distribution of ATAB isolates.** The map illustrates the prevalence of ATAB isolates across various countries, with shading intensity indicating the proportion of these isolates.


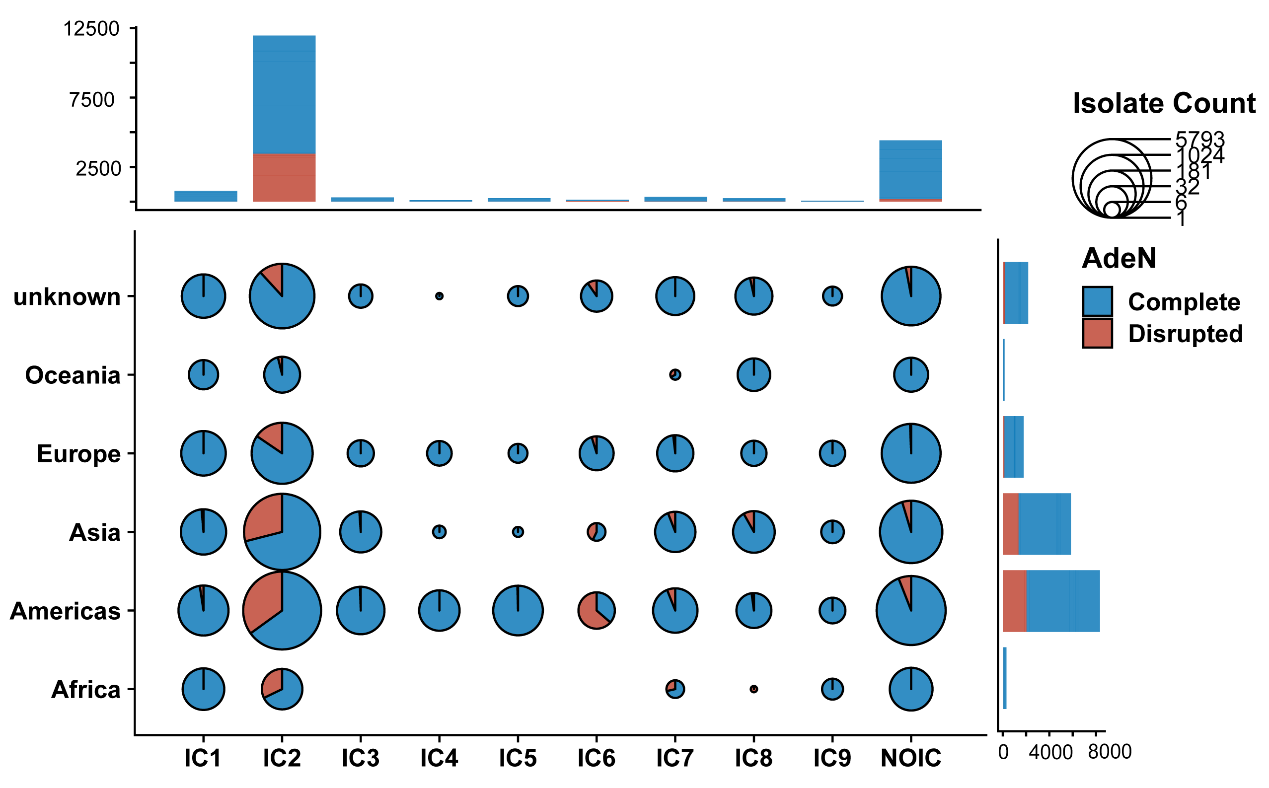


**Figure S3. Distribution of International Clone (IC) among *adeN*-truncated and *adeN*-complete isolates across different continents.** Bubble sizes represent the number of strains, with pie chart segments indicating the proportions of truncated versus complete *adeN* strains.


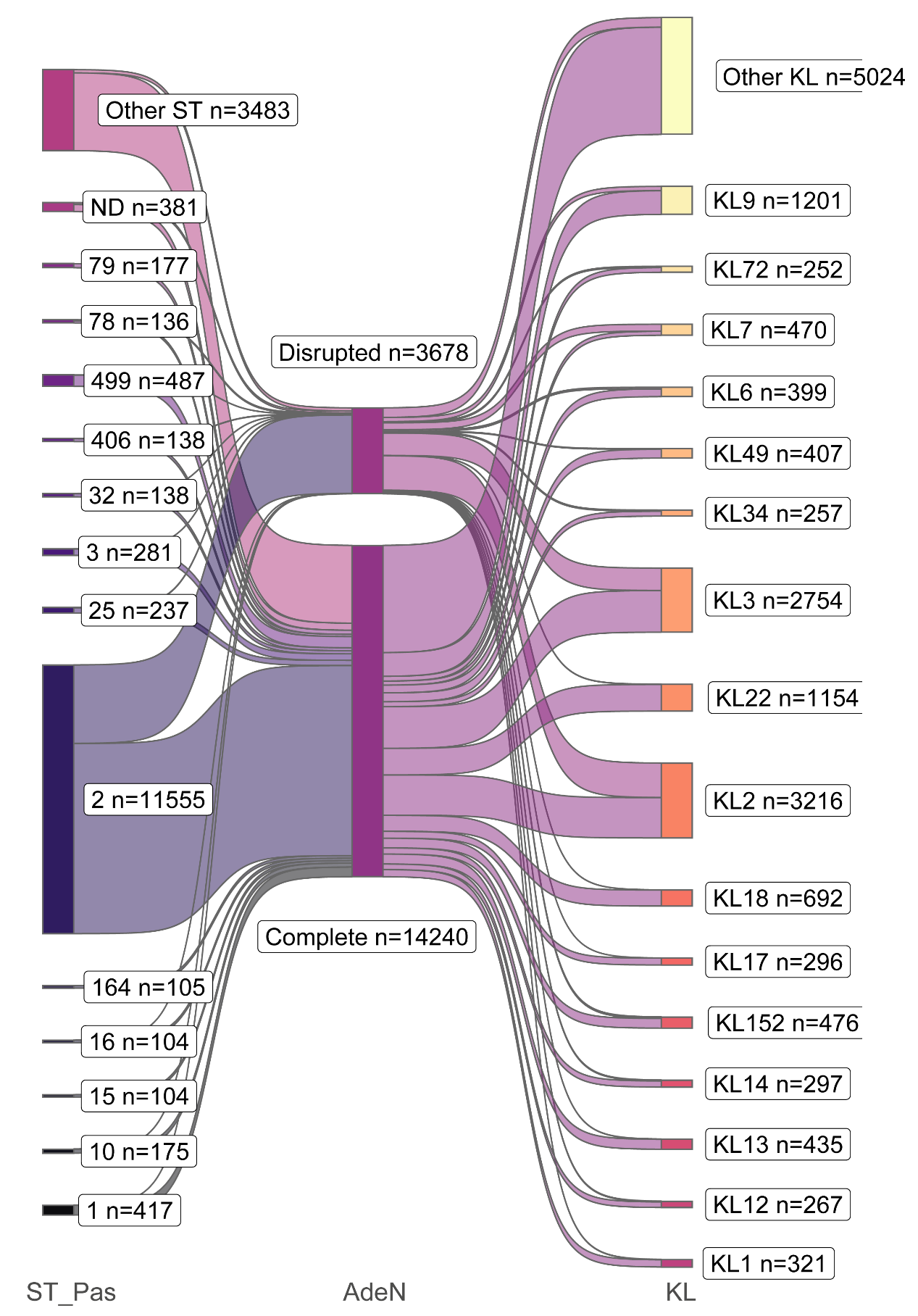


**Figure S4. Sequence type and KL type distribution of *adeN*-truncated and *adeN*-complete *Acinetobacter baumannii* genomes in Genbank.**


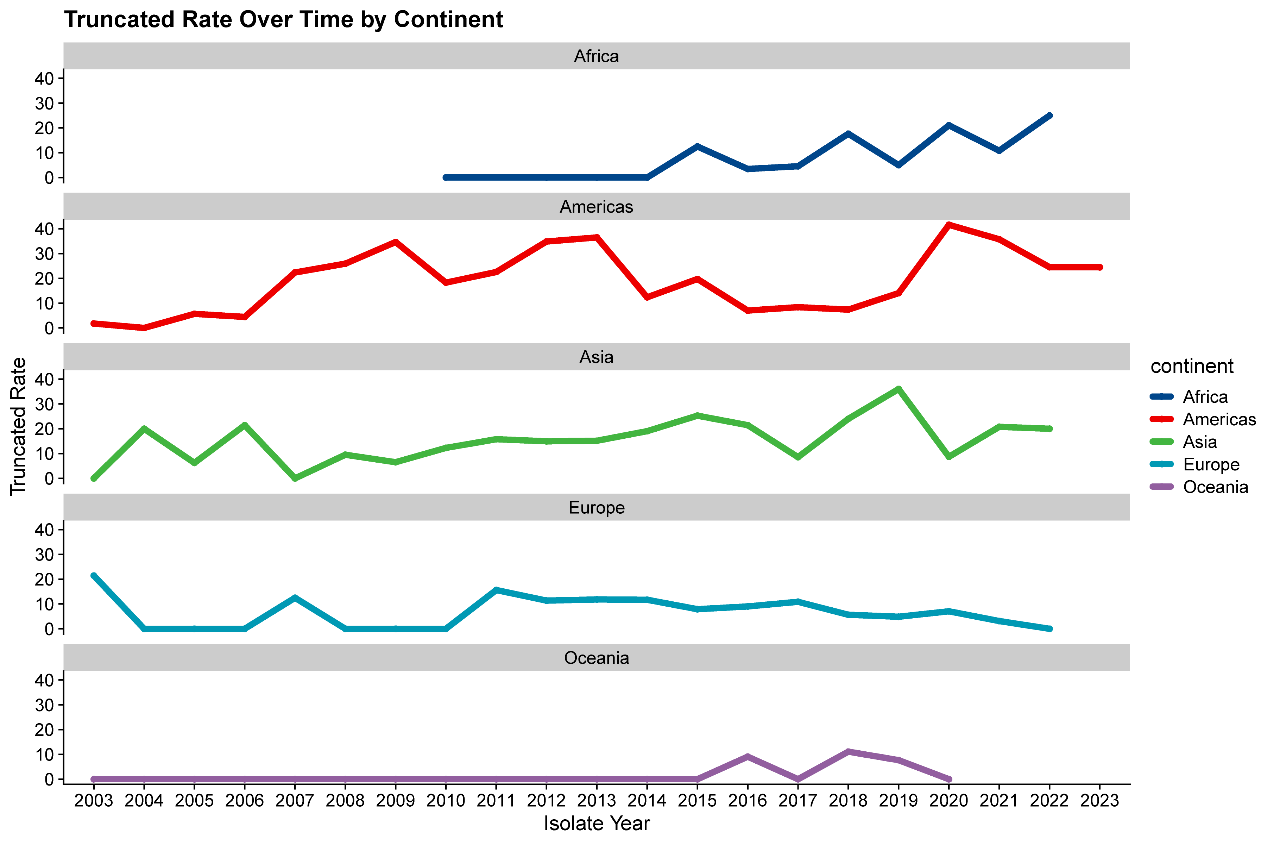


**Figure S5. *adeN*-truncated rate in *Acinetobacter baumannii* across different continents from 2003 to 2023.**

**
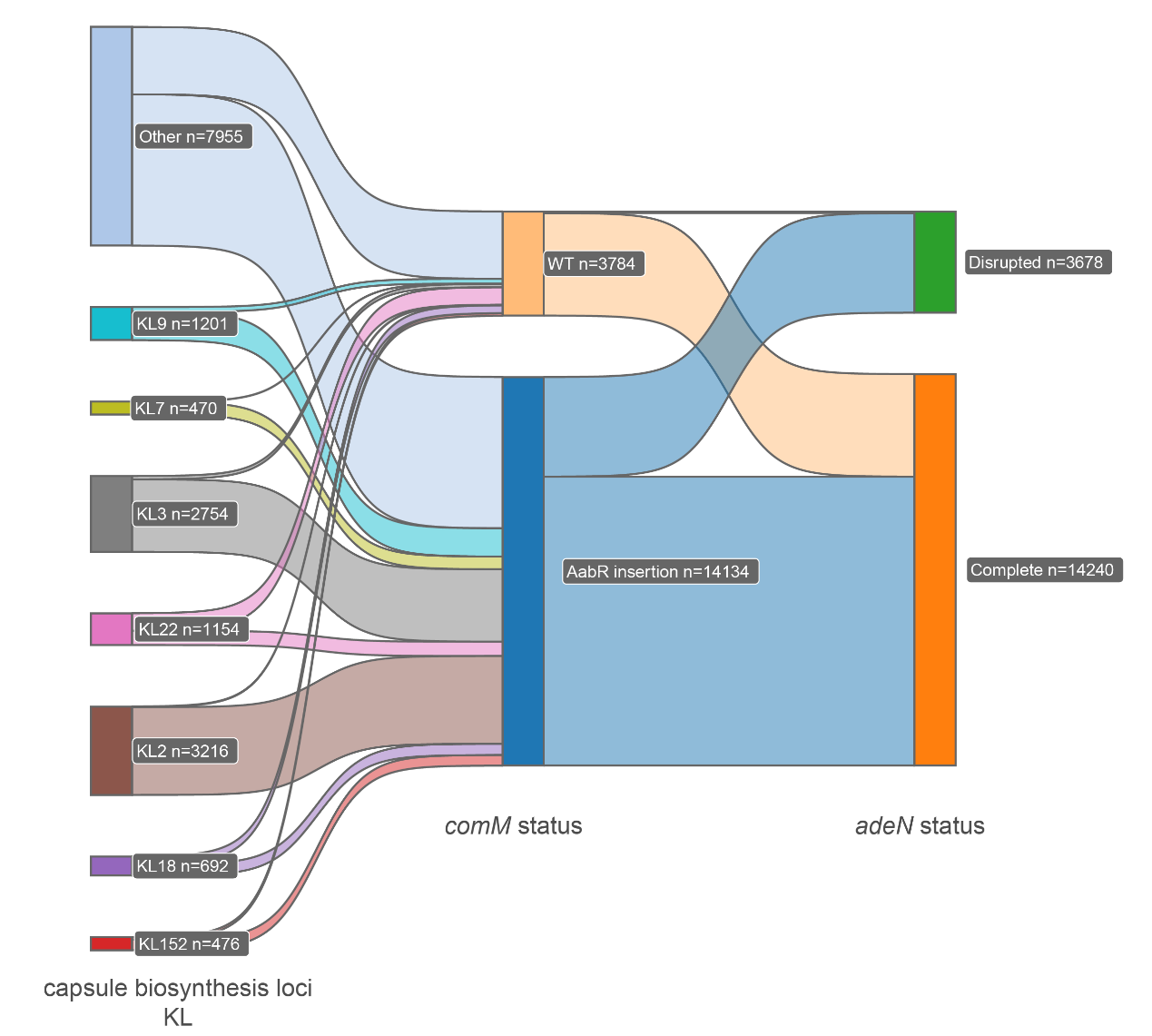
**

**Figure S6. KL type and *comM* status distribution of *adeN*-truncated and *adeN*-complete *Acinetobacter baumannii* genomes in Genbank.**

Table S1 metadata of sequenced ***adeN*-truncated and *adeN*-complete *Acinetobacter baumannii* in this study**

| **ID** | **AdeN** | **Department** | **Sample_Type** | **Year** | **ST_Pas** | **KL** | **ST_Oxf** |
| --- | --- | --- | --- | --- | --- | --- | --- |
| BM6367 | Complete | Surgery department | Drainage fluid | 2020 | ST2 | K7 | 208 |
| BM6369 | Complete | ICU | Urine | 2020 | ST2 | K7 | 208 |
| BM6381 | Complete | Surgery department | Urine | 2020 | Others | Others | Others |
| BM6411 | Complete | Surgery department | Wounds | 2020 | ST2 | Others | Others |
| BM6427 | Complete | Rehabilitation department | Urine | 2020 | ST2 | K7 | 208 |
| BM6449 | Complete | ICU | Drainage fluid | 2020 | ST40 | Others | Others |
| BM6473 | Complete | Surgery department | Wounds | 2020 | Others | K24-Wzy-GI2 | Others |
| BM6485 | Complete | Surgery department | Catheters | 2020 | ST2 | K7 | 208 |
| BM6487 | Complete | Others | Wounds | 2020 | Others | unknown | unknown |
| BM6521 | Complete | RICU | Sputum | 2020 | ST2 | unknown | Others |
| BM6587 | Complete | Burn center | Blood | 2020 | ST2 | unknown | Others |
| BM6601 | Complete | ICU | Wounds | 2020 | ST2 | K7 | 208 |
| BM6605 | Complete | Internal medicine department | Blood | 2020 | Others | unknown | Others |
| BM6611 | Complete | Surgery department | Tissues | 2020 | ST2 | K7 | 208 |
| BM6633 | Complete | Surgery department | Cerebrospinal fluid | 2020 | ST2 | K7 | 208 |
| BM6647 | Complete | Surgery department | Hydrothorax and ascite | 2020 | Others | unknown | unknown |
| BM6653 | Complete | Surgery department | Drainage fluid | 2020 | ST2 | K7 | 208 |
| BM6675 | Complete | Surgery department | Wounds | 2020 | Others | unknown | Others |
| BM6773 | Complete | Surgery department | Blood | 2020 | ST2 | K7 | 208 |
| BM6885 | Complete | ICU | Hydrothorax and ascite | 2020 | ST2 | K7 | 208 |
| BM6899 | Complete | Burn center | Wounds | 2020 | ST2 | K7 | 208 |
| BM6903 | Complete | ICU | Blood | 2020 | ST2 | K7 | 208 |
| BM6915 | Complete | Burn center | Wounds | 2020 | Others | K24-Wzy-GI2 | Others |
| BM6933 | Complete | Surgery department | Blood | 2020 | ST2 | K7 | 208 |
| BM6937 | Complete | Surgery department | Blood | 2020 | Others | unknown | Others |
| BM6953 | Complete | Internal medicine department | Drainage fluid | 2020 | Others | K24-Wzy-GI2 | Others |
| BM6963 | Complete | Surgery department | Cerebrospinal fluid | 2020 | ST2 | K7 | 208 |
| BM6975 | Complete | Internal medicine department | Urine | 2020 | ST2 | K7 | 208 |
| BM7019 | Complete | Burn center | Wounds | 2020 | ST2 | K7 | 208 |
| BM7027 | Complete | ICU | Blood | 2020 | Others | unknown | Others |
| BM7035 | Complete | Surgery department | Catheters | 2020 | ST2 | K7 | 208 |
| BM7045 | Complete | Surgery department | Cerebrospinal fluid | 2020 | ST2 | K7 | 208 |
| BM7055 | Complete | Surgery department | Urine | 2020 | ST40 | Others | Others |
| BM7101 | Complete | Surgery department | Urine | 2020 | ST2 | K7 | 208 |
| BM7129 | Complete | Surgery department | Wounds | 2020 | ST2 | K7 | 208 |
| BM7143 | Complete | ICU | Wounds | 2021 | ST2 | K7 | 208 |
| BM7151 | Complete | Surgery department | Wounds | 2021 | ST2 | K7 | 208 |
| BM7187 | Complete | Surgery department | Wounds | 2021 | ST2 | K7 | 208 |
| BM7189 | Complete | ICU | Catheters | 2021 | ST2 | K7 | 208 |
| BM7197 | Complete | ICU | Wounds | 2021 | ST2 | K7 | 208 |
| BM7199 | Complete | ICU | Cerebrospinal fluid | 2021 | ST2 | K7 | 208 |
| BM7233 | Complete | Surgery department | Sputum | 2021 | ST2 | K7 | 208 |
| BM7241 | Complete | ICU | Blood | 2021 | ST2 | K7 | 208 |
| BM7251 | Complete | RICU | Catheters | 2021 | ST2 | K7 | 208 |
| BM7267 | Complete | ICU | Blood | 2021 | ST2 | K7 | 208 |
| BM7273 | Complete | Surgery department | Wounds | 2021 | Others | Others | Others |
| BM7275 | Complete | Surgery department | Tissues | 2021 | ST2 | K7 | 208 |
| BM7277 | Complete | Rehabilitation department | Urine | 2021 | ST2 | K7 | 208 |
| BM7305 | Complete | CCU | Wounds | 2021 | ST2 | K7 | 208 |
| BM7327 | Complete | ICU | Blood | 2021 | ST2 | K7 | 208 |
| BM7331 | Complete | Surgery department | Urine | 2021 | Others | unknown | Others |
| BM7349 | Complete | ICU | Drainage fluid | 2021 | ST2 | K7 | 208 |
| BM7373 | Complete | ICU | Cerebrospinal fluid | 2021 | ST2 | K7 | 208 |
| BM7381 | Complete | Surgery department | Tissues | 2021 | Others | unknown | Others |
| BM7389 | Complete | Rehabilitation department | Urine | 2021 | ST2 | K7 | 208 |
| BM7407 | Complete | Internal medicine department | Blood | 2021 | ST2 | K7 | 208 |
| BM7419 | Complete | ICU | Cerebrospinal fluid | 2021 | ST2 | K7 | 208 |
| BM7453 | Complete | ICU | Blood | 2021 | ST2 | K7 | 208 |
| BM7469 | Complete | Emergency department | Wounds | 2021 | ST2 | K7 | 208 |
| BM7493 | Complete | ICU | Urine | 2021 | ST2 | K7 | 208 |
| BM7499 | Complete | RICU | Blood | 2021 | ST2 | K7 | 208 |
| BM7517 | Complete | Surgery department | Urine | 2021 | ST2 | K7 | 208 |
| BM7521 | Complete | Surgery department | Urine | 2021 | Others | Others | Others |
| BM7525 | Complete | Surgery department | Urine | 2021 | Others | Others | Others |
| BM7537 | Complete | Rehabilitation department | Urine | 2021 | Others | Others | unknown |
| BM7583 | Complete | ICU | Blood | 2021 | ST2 | K7 | 208 |
| BM7591 | Complete | Internal medicine department | Urine | 2021 | ST2 | K7 | 208 |
| BM7597 | Complete | ICU | Blood | 2021 | ST2 | K7 | 208 |
| BM7615 | Complete | Surgery department | Urine | 2021 | Others | Others | unknown |
| BM7619 | Complete | ICU | Drainage fluid | 2021 | ST2 | K7 | unknown |
| BM7625 | Complete | Surgery department | Wounds | 2021 | Others | unknown | Others |
| BM7633 | Complete | ICU | Wounds | 2021 | ST2 | K7 | 208 |
| BM7637 | Complete | RICU | Blood | 2021 | ST2 | K7 | 208 |
| BM7645 | Complete | Surgery department | Drainage fluid | 2021 | ST2 | K7 | 208 |
| BM7669 | Complete | ICU | Drainage fluid | 2021 | ST2 | K7 | 208 |
| BM7681 | Complete | Surgery department | Urine | 2021 | Others | unknown | unknown |
| BM7683 | Complete | Surgery department | Hydrothorax and ascite | 2021 | ST2 | K7 | 208 |
| BM7695 | Complete | ICU | Blood | 2021 | ST2 | K7 | 208 |
| BM7701 | Complete | Burn center | Wounds | 2021 | ST2 | unknown | 136 |
| BM7719 | Complete | ICU | Wounds | 2021 | ST2 | K7 | 208 |
| BM7751 | Complete | EICU | Blood | 2021 | ST2 | K7 | 208 |
| BM7781 | Complete | EICU | Blood | 2021 | ST2 | K7 | 208 |
| BM7791 | Complete | ICU | Blood | 2021 | ST2 | K7 | 208 |
| BM7803 | Complete | Surgery department | Wounds | 2021 | Others | unknown | unknown |
| BM7811 | Complete | Surgery department | Drainage fluid | 2021 | ST2 | K7 | 208 |
| BM7827 | Complete | Surgery department | Wounds | 2021 | ST2 | K7 | 208 |
| BM7831 | Complete | Surgery department | Urine | 2021 | Others | unknown | unknown |
| BM7837 | Complete | Surgery department | Urine | 2021 | Others | Others | unknown |
| BM7845 | Complete | ICU | Drainage fluid | 2021 | ST2 | K7 | 208 |
| BM7849 | Complete | Surgery department | Drainage fluid | 2021 | Others | Others | unknown |
| BM7869 | Complete | Surgery department | Drainage fluid | 2021 | Others | K24-Wzy-GI2 | Others |
| BM7877 | Complete | Surgery department | Cerebrospinal fluid | 2021 | ST2 | K7 | 208 |
| BM7899 | Complete | ICU | Cerebrospinal fluid | 2021 | ST2 | K7 | 208 |
| BM7907 | Complete | Surgery department | Tissues | 2022 | ST2 | K7 | 208 |
| BM7925 | Complete | ICU | Drainage fluid | 2022 | ST2 | K7 | 208 |
| BM7951 | Complete | RICU | Blood | 2022 | ST2 | Others | Others |
| BM7987 | Complete | Surgery department | Urine | 2022 | Others | Others | Others |
| BM8021 | Complete | Internal medicine department | Hydrothorax and ascite | 2022 | Others | K7 | 208 |
| BM8031 | Complete | EICU | Blood | 2022 | ST2 | unknown | Others |
| BM8051 | Complete | ICU | Wounds | 2022 | ST2 | K7 | unknown |
| BM8057 | Complete | Surgery department | Wounds | 2022 | ST2 | K7 | 208 |
| BM8077 | Complete | Infectious disease department | Urine | 2022 | ST2 | K7 | 208 |
| BM8103 | Complete | Surgery department | Wounds | 2022 | Others | Others | unknown |
| BM8121 | Complete | Surgery department | Tissues | 2022 | ST2 | K7 | 208 |
| BM8137 | Complete | Surgery department | Catheters | 2022 | ST2 | K7 | 208 |
| BM8145 | Complete | Internal medicine department | Blood | 2022 | ST40 | Others | Others |
| BM8147 | Complete | Surgery department | Wounds | 2022 | Others | unknown | unknown |
| BM8157 | Complete | ICU | Blood | 2022 | ST2 | K7 | 208 |
| BM8165 | Complete | ICU | Drainage fluid | 2022 | ST2 | K7 | 208 |
| BM8187 | Complete | Surgery department | Urine | 2022 | Others | Others | unknown |
| BM8227 | Complete | RICU | Sputum | 2022 | ST2 | K7 | 208 |
| BM8249 | Complete | Emergency department | Blood | 2022 | ST2 | K7 | 208 |
| BM8257 | Complete | Internal medicine department | Hydrothorax and ascite | 2022 | Others | Others | unknown |
| BM8279 | Complete | Surgery department | Wounds | 2022 | ST2 | K7 | 208 |
| BM8287 | Complete | RICU | Urine | 2022 | ST2 | K7 | 208 |
| BM8319 | Complete | ICU | Catheters | 2022 | ST2 | K7 | unknown |
| BM8339 | Complete | ICU | Blood | 2022 | ST2 | K7 | unknown |
| BM8457 | Complete | Surgery department | Catheters | 2022 | ST2 | K7 | 208 |
| BM8497 | Complete | Surgery department | Tissues | 2022 | Others | unknown | unknown |
| BM6355 | Truncated | ICU | Sputum | 2020 | ST2 | K2 | 208 |
| BM6359 | Truncated | RICU | Hydrothorax and ascite | 2020 | ST2 | K2 | 208 |
| BM6371 | Truncated | Infectious disease department | Urine | 2020 | ST2 | K2 | 208 |
| BM6373 | Truncated | ICU | Sputum | 2020 | ST2 | K2 | 208 |
| BM6377 | Truncated | ICU | Sputum | 2020 | ST2 | K2 | 208 |
| BM6383 | Truncated | EICU | Sputum | 2020 | ST2 | K2 | 208 |
| BM6385 | Truncated | ICU | Sputum | 2020 | ST2 | K2 | 208 |
| BM6391 | Truncated | ICU | Sputum | 2020 | ST2 | K2 | 208 |
| BM6393 | Truncated | Internal medicine department | Hydrothorax and ascite | 2020 | ST2 | K2 | 208 |
| BM6395 | Truncated | EICU | Sputum | 2020 | ST2 | K2 | 208 |
| BM6397 | Truncated | ICU | Sputum | 2020 | ST2 | K2 | 208 |
| BM6401 | Truncated | RICU | Sputum | 2020 | ST2 | K2 | 208 |
| BM6403 | Truncated | ICU | Sputum | 2020 | ST2 | K2 | 208 |
| BM6407 | Truncated | EICU | Sputum | 2020 | ST2 | K2 | 208 |
| BM6417 | Truncated | ICU | Sputum | 2020 | ST2 | K2 | 208 |
| BM6419 | Truncated | RICU | Sputum | 2020 | ST2 | K2 | 208 |
| BM6421 | Truncated | CCU | Sputum | 2020 | ST2 | K2 | 208 |
| BM6423 | Truncated | ICU | Catheters | 2020 | ST2 | K2 | 208 |
| BM6431 | Truncated | ICU | Sputum | 2020 | ST2 | K2 | 208 |
| BM6433 | Truncated | EICU | Sputum | 2020 | ST2 | K2 | 208 |
| BM6435 | Truncated | EICU | Sputum | 2020 | ST2 | K2 | 208 |
| BM6437 | Truncated | Emergency department | Others | 2020 | ST2 | K2 | 208 |
| BM6439 | Truncated | EICU | Sputum | 2020 | ST2 | K2 | 208 |
| BM6447 | Truncated | Surgery department | Sputum | 2020 | ST2 | Others | Others |
| BM6451 | Truncated | ICU | Drainage fluid | 2020 | ST2 | K2 | 208 |
| BM6453 | Truncated | RICU | Sputum | 2020 | ST2 | K2 | 208 |
| BM6467 | Truncated | EICU | Sputum | 2020 | ST2 | K2 | 208 |
| BM6471 | Truncated | EICU | Sputum | 2020 | ST2 | K2 | 208 |
| BM6475 | Truncated | RICU | Sputum | 2020 | ST2 | K2 | 208 |
| BM6479 | Truncated | EICU | Sputum | 2020 | ST2 | K2 | 208 |
| BM6481 | Truncated | Surgery department | Sputum | 2020 | ST2 | K2 | 208 |
| BM6489 | Truncated | ICU | Sputum | 2020 | ST2 | K2 | 208 |
| BM6493 | Truncated | Others | Wounds | 2020 | ST2 | K2 | 208 |
| BM6495 | Truncated | EICU | Sputum | 2020 | ST2 | K2 | 208 |
| BM6499 | Truncated | RICU | Sputum | 2020 | ST2 | K2 | 208 |
| BM6501 | Truncated | ICU | Sputum | 2020 | ST2 | K2 | 208 |
| BM6503 | Truncated | CCU | Sputum | 2020 | ST2 | K2 | 208 |
| BM6505 | Truncated | EICU | Sputum | 2020 | ST2 | K2 | 208 |
| BM6507 | Truncated | Surgery department | Sputum | 2020 | ST2 | K2 | 208 |
| BM6511 | Truncated | ICU | Sputum | 2020 | ST2 | K2 | 208 |
| BM6513 | Truncated | Internal medicine department | Sputum | 2020 | ST2 | K2 | 208 |
| BM6515 | Truncated | RICU | Sputum | 2020 | ST2 | K2 | 208 |
| BM6517 | Truncated | ICU | Sputum | 2020 | ST2 | K2 | 208 |
| BM6531 | Truncated | RICU | Sputum | 2020 | ST2 | K2 | 208 |
| BM6545 | Truncated | Surgery department | Blood | 2020 | ST2 | K2 | 208 |
| BM6547 | Truncated | Surgery department | Sputum | 2020 | ST2 | K2 | 208 |
| BM6551 | Truncated | ICU | Sputum | 2020 | ST2 | K2 | 208 |
| BM6553 | Truncated | Internal medicine department | Sputum | 2020 | ST2 | K2 | 208 |
| BM6557 | Truncated | Surgery department | Blood | 2020 | ST2 | K2 | 208 |
| BM6559 | Truncated | ICU | Sputum | 2020 | ST2 | K2 | 208 |
| BM6563 | Truncated | Infectious disease department | Urine | 2020 | ST2 | K2 | 208 |
| BM6567 | Truncated | RICU | Sputum | 2020 | ST2 | K2 | 208 |
| BM6569 | Truncated | ICU | Sputum | 2020 | ST2 | K2 | 208 |
| BM6573 | Truncated | Surgery department | Sputum | 2020 | ST2 | K2 | 208 |
| BM6575 | Truncated | RICU | Sputum | 2020 | ST2 | K2 | 208 |
| BM6579 | Truncated | Burn center | Tissues | 2020 | ST2 | K2 | 208 |
| BM6581 | Truncated | ICU | Sputum | 2020 | ST2 | K2 | 208 |
| BM6583 | Truncated | EICU | Sputum | 2020 | ST2 | K2 | 208 |
| BM6597 | Truncated | EICU | Sputum | 2020 | ST2 | K2 | 208 |
| BM6599 | Truncated | ICU | Sputum | 2020 | ST2 | K2 | 208 |
| BM6609 | Truncated | Surgery department | Sputum | 2020 | ST2 | K2 | 208 |
| BM6613 | Truncated | Burn center | Sputum | 2020 | ST2 | K2 | 208 |
| BM6615 | Truncated | ICU | Wounds | 2020 | ST2 | K2 | 208 |
| BM6623 | Truncated | ICU | Sputum | 2020 | ST2 | K2 | 208 |
| BM6625 | Truncated | Surgery department | Sputum | 2020 | ST2 | K2 | 208 |
| BM6631 | Truncated | Surgery department | Sputum | 2020 | ST2 | K2 | 208 |
| BM6635 | Truncated | ICU | Sputum | 2020 | ST2 | K2 | 208 |
| BM6643 | Truncated | Surgery department | Sputum | 2020 | ST2 | K2 | 208 |
| BM6657 | Truncated | Surgery department | Sputum | 2020 | ST2 | K2 | 208 |
| BM6659 | Truncated | Surgery department | Sputum | 2020 | ST2 | K2 | 208 |
| BM6669 | Truncated | ICU | Sputum | 2020 | ST2 | K2 | 208 |
| BM6671 | Truncated | ICU | Sputum | 2020 | ST2 | K2 | 208 |
| BM6677 | Truncated | ICU | Sputum | 2020 | ST2 | K2 | 208 |
| BM6679 | Truncated | ICU | Sputum | 2020 | ST2 | K2 | 208 |
| BM6683 | Truncated | Surgery department | Drainage fluid | 2020 | ST2 | K2 | 208 |
| BM6695 | Truncated | ICU | Sputum | 2020 | ST2 | K2 | 208 |
| BM6697 | Truncated | RICU | Sputum | 2020 | ST2 | K2 | 208 |
| BM6701 | Truncated | ICU | Sputum | 2020 | ST2 | K2 | 208 |
| BM6713 | Truncated | Surgery department | Sputum | 2020 | ST2 | K2 | 208 |
| BM6717 | Truncated | Burn center | Catheters | 2020 | ST2 | K2 | 208 |
| BM6731 | Truncated | ICU | Sputum | 2020 | ST2 | K2 | 208 |
| BM6735 | Truncated | Surgery department | Sputum | 2020 | ST2 | K2 | 208 |
| BM6743 | Truncated | Surgery department | Sputum | 2020 | ST2 | K2 | 208 |
| BM6745 | Truncated | ICU | Sputum | 2020 | ST2 | K2 | 208 |
| BM6753 | Truncated | Burn center | Wounds | 2020 | ST2 | K2 | 208 |
| BM6755 | Truncated | ICU | Sputum | 2020 | ST2 | K2 | 208 |
| BM6757 | Truncated | EICU | Sputum | 2020 | ST2 | K2 | 208 |
| BM6767 | Truncated | Surgery department | Sputum | 2020 | ST2 | K2 | 208 |
| BM6771 | Truncated | Surgery department | Sputum | 2020 | ST2 | K2 | 208 |
| BM6775 | Truncated | ICU | Sputum | 2020 | ST2 | K2 | 208 |
| BM6781 | Truncated | ICU | Sputum | 2020 | ST2 | K2 | 208 |
| BM6787 | Truncated | EICU | Sputum | 2020 | ST2 | K2 | 208 |
| BM6789 | Truncated | EICU | Sputum | 2020 | ST2 | K2 | 208 |
| BM6793 | Truncated | Surgery department | Sputum | 2020 | ST2 | K2 | 208 |
| BM6795 | Truncated | ICU | Sputum | 2020 | ST2 | K2 | 208 |
| BM6797 | Truncated | Surgery department | Sputum | 2020 | ST2 | K2 | 208 |
| BM6801 | Truncated | EICU | Sputum | 2020 | ST2 | K2 | 208 |
| BM6807 | Truncated | Surgery department | Sputum | 2020 | ST2 | K2 | 208 |
| BM6809 | Truncated | ICU | Sputum | 2020 | ST2 | K2 | 208 |
| BM6815 | Truncated | Surgery department | Sputum | 2020 | ST2 | K2 | 208 |
| BM6817 | Truncated | ICU | Sputum | 2020 | ST2 | K2 | 208 |
| BM6825 | Truncated | Surgery department | Urine | 2020 | ST2 | K2 | 208 |
| BM6837 | Truncated | ICU | Sputum | 2020 | ST2 | K2 | 208 |
| BM6839 | Truncated | ICU | Sputum | 2020 | ST2 | K2 | 208 |
| BM6847 | Truncated | ICU | Sputum | 2020 | ST2 | K2 | 208 |
| BM6859 | Truncated | Internal medicine department | Sputum | 2020 | ST2 | K2 | 208 |
| BM6861 | Truncated | ICU | Sputum | 2020 | ST2 | K2 | 208 |
| BM6863 | Truncated | Surgery department | Drainage fluid | 2020 | ST2 | K7 | 208 |
| BM6879 | Truncated | RICU | Sputum | 2020 | ST2 | K2 | 208 |
| BM6883 | Truncated | Rehabilitation department | Wounds | 2020 | ST2 | K2 | 208 |
| BM6891 | Truncated | Surgery department | Sputum | 2020 | ST2 | K2 | 208 |
| BM6895 | Truncated | ICU | Sputum | 2020 | ST2 | K2 | 208 |
| BM6901 | Truncated | Surgery department | Drainage fluid | 2020 | ST2 | K2 | 208 |
| BM6907 | Truncated | EICU | Wounds | 2020 | ST2 | Others | Others |
| BM6909 | Truncated | Surgery department | Sputum | 2020 | ST2 | K2 | 208 |
| BM6913 | Truncated | Burn center | Sputum | 2020 | ST2 | K2 | 208 |
| BM6921 | Truncated | ICU | Sputum | 2020 | ST2 | K7 | 208 |
| BM6927 | Truncated | EICU | Sputum | 2020 | ST2 | K2 | 208 |
| BM6929 | Truncated | EICU | Sputum | 2020 | ST2 | K2 | 208 |
| BM6935 | Truncated | EICU | Sputum | 2020 | ST2 | K2 | 208 |
| BM6943 | Truncated | ICU | Sputum | 2020 | ST2 | K2 | 208 |
| BM6949 | Truncated | Burn center | Wounds | 2020 | ST2 | Others | Others |
| BM6955 | Truncated | ICU | Wounds | 2020 | ST2 | K7 | 208 |
| BM6961 | Truncated | Burn center | Tissues | 2020 | ST2 | Others | Others |
| BM6973 | Truncated | ICU | Sputum | 2020 | ST2 | K2 | 208 |
| BM6983 | Truncated | ICU | Sputum | 2020 | ST2 | K2 | 208 |
| BM6985 | Truncated | Surgery department | Urine | 2020 | Others | Others | Others |
| BM6989 | Truncated | EICU | Sputum | 2020 | ST2 | K2 | 208 |
| BM7001 | Truncated | EICU | Sputum | 2020 | ST2 | K2 | 208 |
| BM7011 | Truncated | Surgery department | Sputum | 2020 | ST2 | K2 | 208 |
| BM7039 | Truncated | ICU | Sputum | 2020 | ST2 | K2 | 208 |
| BM7063 | Truncated | Surgery department | Urine | 2020 | ST2 | K2 | 208 |
| BM7065 | Truncated | Surgery department | Sputum | 2020 | ST2 | K2 | 208 |
| BM7083 | Truncated | RICU | Sputum | 2020 | ST2 | K2 | 208 |
| BM7093 | Truncated | Rehabilitation department | Sputum | 2020 | ST2 | K2 | 208 |
| BM7111 | Truncated | ICU | Sputum | 2020 | ST2 | K2 | 208 |
| BM7115 | Truncated | Surgery department | Sputum | 2020 | ST2 | K2 | 208 |
| BM7121 | Truncated | ICU | Drainage fluid | 2020 | ST2 | K2 | 208 |
| BM7161 | Truncated | ICU | Sputum | 2021 | ST2 | K2 | 208 |
| BM7163 | Truncated | ICU | Sputum | 2021 | ST2 | K2 | 208 |
| BM7167 | Truncated | ICU | Sputum | 2021 | ST2 | K2 | 208 |
| BM7173 | Truncated | ICU | Sputum | 2021 | ST2 | K2 | 208 |
| BM7175 | Truncated | ICU | Sputum | 2021 | ST2 | K2 | 208 |
| BM7179 | Truncated | RICU | Sputum | 2021 | ST2 | K2 | 208 |
| BM7181 | Truncated | ICU | Sputum | 2021 | ST2 | K2 | 208 |
| BM7203 | Truncated | RICU | Sputum | 2021 | ST2 | K2 | 208 |
| BM7215 | Truncated | ICU | Sputum | 2021 | ST2 | K2 | 208 |
| BM7217 | Truncated | ICU | Sputum | 2021 | ST2 | K2 | 208 |
| BM7229 | Truncated | ICU | Sputum | 2021 | ST2 | K2 | 208 |
| BM7237 | Truncated | ICU | Sputum | 2021 | ST2 | K2 | 208 |
| BM7247 | Truncated | ICU | Sputum | 2021 | ST2 | K2 | 208 |
| BM7249 | Truncated | RICU | Sputum | 2021 | ST2 | K2 | 208 |
| BM7253 | Truncated | ICU | Sputum | 2021 | ST2 | K2 | 208 |
| BM7259 | Truncated | ICU | Sputum | 2021 | ST2 | K2 | 208 |
| BM7265 | Truncated | RICU | Sputum | 2021 | ST2 | K2 | 208 |
| BM7279 | Truncated | ICU | Sputum | 2021 | ST2 | K2 | 208 |
| BM7283 | Truncated | ICU | Sputum | 2021 | ST2 | K2 | 208 |
| BM7285 | Truncated | ICU | Sputum | 2021 | ST2 | K2 | 208 |
| BM7287 | Truncated | ICU | Sputum | 2021 | ST2 | K2 | 208 |
| BM7293 | Truncated | ICU | Sputum | 2021 | ST2 | K2 | 208 |
| BM7295 | Truncated | Internal medicine department | Sputum | 2021 | ST2 | K2 | 208 |
| BM7309 | Truncated | CCU | Sputum | 2021 | ST2 | K2 | 208 |
| BM7313 | Truncated | ICU | Sputum | 2021 | ST2 | K2 | 208 |
| BM7319 | Truncated | RICU | Sputum | 2021 | ST2 | K2 | 208 |
| BM7325 | Truncated | ICU | Sputum | 2021 | ST2 | K2 | 208 |
| BM7335 | Truncated | Surgery department | Sputum | 2021 | ST2 | K2 | 208 |
| BM7337 | Truncated | ICU | Sputum | 2021 | ST2 | K2 | 208 |
| BM7339 | Truncated | ICU | Sputum | 2021 | ST2 | K2 | 208 |
| BM7343 | Truncated | ICU | Sputum | 2021 | ST2 | K2 | 208 |
| BM7345 | Truncated | ICU | Blood | 2021 | ST2 | K2 | 208 |
| BM7351 | Truncated | ICU | Sputum | 2021 | ST2 | K2 | 208 |
| BM7361 | Truncated | ICU | Sputum | 2021 | ST2 | K2 | 208 |
| BM7365 | Truncated | ICU | Sputum | 2021 | ST2 | K2 | 208 |
| BM7369 | Truncated | Surgery department | Sputum | 2021 | ST2 | K2 | 208 |
| BM7371 | Truncated | ICU | Sputum | 2021 | ST2 | K2 | 208 |
| BM7395 | Truncated | ICU | Cerebrospinal fluid | 2021 | ST2 | K2 | 208 |
| BM7405 | Truncated | ICU | Sputum | 2021 | ST2 | K2 | 208 |
| BM7411 | Truncated | ICU | Hydrothorax and ascite | 2021 | ST2 | K2 | 208 |
| BM7421 | Truncated | ICU | Blood | 2021 | ST2 | Others | Others |
| BM7431 | Truncated | ICU | Sputum | 2021 | ST2 | K2 | 208 |
| BM7467 | Truncated | ICU | Sputum | 2021 | ST2 | K2 | 208 |
| BM7475 | Truncated | ICU | Sputum | 2021 | ST2 | K7 | 208 |
| BM7481 | Truncated | Burn center | Sputum | 2021 | ST2 | K2 | 208 |
| BM7487 | Truncated | Internal medicine department | Bronchoalveolar lavage fluid | 2021 | ST2 | K2 | 208 |
| BM7509 | Truncated | ICU | Hydrothorax and ascite | 2021 | ST2 | K2 | 208 |
| BM7531 | Truncated | ICU | Sputum | 2021 | ST2 | K2 | 208 |
| BM7621 | Truncated | ICU | Sputum | 2021 | ST2 | K2 | 208 |
| BM7641 | Truncated | ICU | Sputum | 2021 | ST2 | K2 | 208 |
| BM7657 | Truncated | Internal medicine department | Sputum | 2021 | ST2 | K2 | 208 |
| BM7659 | Truncated | RICU | Sputum | 2021 | ST2 | K2 | 208 |
| BM7671 | Truncated | EICU | Sputum | 2021 | ST2 | K2 | 208 |
| BM7673 | Truncated | RICU | Bronchoalveolar lavage fluid | 2021 | ST2 | K2 | 208 |
| BM7715 | Truncated | RICU | Blood | 2021 | ST2 | K2 | 208 |
| BM7717 | Truncated | Internal medicine department | Sputum | 2021 | ST2 | K2 | 208 |
| BM7721 | Truncated | ICU | Sputum | 2021 | ST2 | K2 | 208 |
| BM7737 | Truncated | Internal medicine department | Sputum | 2021 | ST2 | K2 | 208 |
| BM7745 | Truncated | ICU | Sputum | 2021 | ST2 | K7 | 208 |
| BM7755 | Truncated | RICU | Sputum | 2021 | ST2 | K2 | 208 |
| BM7757 | Truncated | ICU | Sputum | 2021 | ST2 | K2 | 208 |
| BM7787 | Truncated | EICU | Sputum | 2021 | ST1555 | K2 | 2211 |
| BM7817 | Truncated | CCU | Blood | 2021 | ST1555 | K2 | 2211 |
| BM8003 | Truncated | RICU | Sputum | 2022 | ST2 | unknown | 136 |
| BM8011 | Truncated | RICU | Blood | 2022 | ST2 | unknown | 136 |
| BM8023 | Truncated | RICU | Sputum | 2022 | ST2 | unknown | 136 |
| BM8033 | Truncated | RICU | Sputum | 2022 | ST2 | K2 | 208 |
| BM8069 | Truncated | ICU | Sputum | 2022 | ST2 | K2 | 208 |
| BM8107 | Truncated | ICU | Sputum | 2022 | ST2 | K2 | 208 |
| BM8111 | Truncated | ICU | Sputum | 2022 | ST2 | K7 | 208 |
| BM8125 | Truncated | ICU | Sputum | 2022 | ST2 | K7 | 208 |
| BM8231 | Truncated | Rehabilitation department | Sputum | 2022 | ST2 | unknown | 136 |
| BM8233 | Truncated | ICU | Sputum | 2022 | ST2 | K7 | 208 |
| BM8243 | Truncated | Surgery department | Wounds | 2022 | ST2 | K7 | 208 |
| BM8283 | Truncated | Surgery department | Sputum | 2022 | ST2 | K7 | 208 |
| BM8299 | Truncated | ICU | Sputum | 2022 | ST2 | K7 | 208 |
| BM8315 | Truncated | Infectious disease department | Sputum | 2022 | ST2 | K2 | 208 |
| BM8325 | Truncated | EICU | Sputum | 2022 | ST2 | K2 | 208 |
| BM8329 | Truncated | EICU | Sputum | 2022 | ST2 | K2 | 208 |
| BM8343 | Truncated | ICU | Sputum | 2022 | ST2 | K2 | 208 |
| BM8345 | Truncated | Surgery department | Tissues | 2022 | ST2 | K7 | 208 |
| BM8359 | Truncated | ICU | Sputum | 2022 | ST2 | K2 | 208 |
| BM8363 | Truncated | Surgery department | Wounds | 2022 | ST2 | K2 | 208 |
| BM8387 | Truncated | ICU | Sputum | 2022 | ST1555 | K2 | 2211 |
| BM8397 | Truncated | ICU | Drainage fluid | 2022 | ST1555 | K2 | 2211 |
| BM8409 | Truncated | Surgery department | Sputum | 2022 | ST2 | K2 | 208 |
| BM8411 | Truncated | ICU | Sputum | 2022 | ST2 | K2 | 208 |
| BM8417 | Truncated | EICU | Sputum | 2022 | ST2 | K2 | 208 |
| BM8423 | Truncated | EICU | Sputum | 2022 | ST2 | K2 | 208 |
| BM8431 | Truncated | ICU | Sputum | 2022 | ST2 | K2 | 208 |
| BM8433 | Truncated | ICU | Sputum | 2022 | ST2 | K2 | 208 |
| BM8439 | Truncated | RICU | Sputum | 2022 | ST2 | K7 | 208 |
| BM8441 | Truncated | Infectious disease department | Sputum | 2022 | ST2 | K2 | 208 |
| BM8443 | Truncated | EICU | Bronchoalveolar lavage fluid | 2022 | ST2 | K2 | 208 |
| BM8451 | Truncated | Surgery department | Sputum | 2022 | ST1555 | K2 | 2211 |
| BM8453 | Truncated | ICU | Sputum | 2022 | ST2 | K2 | 208 |
| BM8455 | Truncated | EICU | Sputum | 2022 | ST2 | K2 | 208 |
| BM8465 | Truncated | Emergency department | Urine | 2022 | ST2 | K2 | 208 |
| BM8475 | Truncated | ICU | Sputum | 2022 | ST1555 | K2 | 2211 |
| BM8477 | Truncated | Internal medicine department | Sputum | 2022 | ST2 | K2 | 208 |
| BM8487 | Truncated | Others | Bronchoalveolar lavage fluid | 2022 | ST1555 | K2 | 2211 |
| BM8507 | Truncated | ICU | Sputum | 2022 | ST2 | K2 | 208 |
| BM8515 | Truncated | Surgery department | Wounds | 2022 | ST2 | K7 | 208 |
| BM8521 | Truncated | Internal medicine department | Sputum | 2022 | ST2 | K7 | 208 |
| BM8523 | Truncated | ICU | Sputum | 2022 | ST2 | K7 | 208 |
| BM8533 | Truncated | ICU | Sputum | 2022 | ST2 | K7 | 208 |
| BM8565 | Truncated | ICU | Sputum | 2022 | ST2 | K2 | 208 |
| BM8575 | Truncated | EICU | Sputum | 2022 | ST2 | K2 | 208 |
| BM8577 | Truncated | EICU | Sputum | 2022 | ST2 | K2 | 208 |
| BM8605 | Truncated | ICU | Sputum | 2022 | ST2 | K7 | 208 |
| BM8609 | Truncated | ICU | Urine | 2022 | ST2 | K7 | 208 |
| BM8611 | Truncated | ICU | Sputum | 2022 | ST1555 | K2 | 2211 |
| BM8617 | Truncated | Surgery department | Wounds | 2022 | ST2 | K2 | 208 |
| BM8625 | Truncated | ICU | Sputum | 2022 | ST2 | K7 | 208 |
| BM8627 | Truncated | Surgery department | Sputum | 2022 | ST2 | K7 | 208 |
| BM8633 | Truncated | EICU | Sputum | 2022 | ST2 | K2 | 208 |

| **Table S2 Demographics and clinical characteristics of patients with** Acinetobacter baumannii **infection.** | | | |
| --- | --- | --- | --- |
| **Variable** | Truncated, N = 611 | Complete, N = 611 | p-value2 |
| **Demographics** |  |  |  |
| Age(years) | 57 (47, 68) | 61 (51, 70) | 0.4 |
| Gender(male) | 15 (24.6%) | 20 (32.8%) | 0.3 |
| Days of hospitalization | 42 (23, 73) | 27 (16, 54) | 0.021 |
| ICU | 46 (75.4%) | 24 (39.3%) | <0.001 |
| Days in ICU | 10 (2, 20) | 0 (0, 16) | 0.005 |
| Days of prior hospitalization | 16 (10, 29) | 10 (5, 23) | 0.008 |
| **Underlying conditions** |  |  |  |
| Charlson comorbidity index | 4.0 (3.0, 7.0) | 4.0 (2.0, 8.0) | 0.8 |
| Diabetes | 5 (8.2%) | 5 (8.2%) | >0.9 |
| Diabetes melitus with end-organ damage | 9 (14.7%) | 6 (9.8%) | 0.4 |
| Chronic liver disease | 6 (9.8%) | 11 (18.0%) | 0.2 |
| Severe liver disease | 18 (29.5%) | 7 (11.5%) | 0.014 |
| Dementia | 1 (1.6%) | 1 (1.6%) | >0.9 |
| Peripheral vascular disease | 10 (16.4%) | 1 (1.6%) | 0.004 |
| Cerebrovascular disease | 9 (14.8%) | 11 (18.0%) | 0.6 |
| Coronary artery disease | 7 (11.5%) | 8 (13.1%) | 0.8 |
| Congestive heart failure | 8 (13.1%) | 7 (11.5%) | 0.8 |
| Renal-replacement therapy | 18 (29.5%) | 14 (23.0%) | 0.4 |
| Chronic obstructive pulmonary disease | 3 (4.9%) | 5 (8.2%) | 0.7 |
| Gastric or peptic ulcers | 1 (1.6%) | 1 (1.6%) | >0.9 |
| Autoimmune disease | 3 (4.9%) | 2 (3.3%) | >0.9 |
| Use of immunosuppressive agent (s) | 10 (16.4%) | 1 (1.6%) | 0.004 |
| Leukaemia | 1 (1.6%) | 1 (1.6%) | >0.9 |
| Solid malignancy | 7 (11.5%) | 19 (31.1%) | 0.008 |
| Metastatic malignancy | 4 (6.6%) | 10 (16.4%) | 0.088 |
| Hypertension | 23 (37.7%) | 25 (41.0%) | 0.7 |
| Endotracheal intubation/tracheotomy | 45 (73.7%) | 27 (44.3%) | <0.001 |
| **Infection source** |  |  |  |
| Pneumonia | 32 (52.5%) | 23 (37.7%) | 0.1 |
| Ventilator-associated pneumonia | 1 (1.6%) | 0 (0%) | >0.9 |
| Catheter-related infection | 3 (4.9%) | 2 (3.3%) | >0.9 |
| Primary bacteraemia | 17 (27.9%) | 14 (23.0%) | 0.7 |
| **Clinical characteristic** |  |  |  |
| White blood cell count (×103 /μL) | 9.9 (6.9, 13.2) | 9.2 (6.9, 12.6) | 0.6 |
| Haemoglobin (g/dL) | 91 (79, 100) | 97 (84, 119) | 0.023 |
| Platelet count (×103/μL) | 212 (131, 359) | 213 (160, 301) | 0.9 |
| Aspartate aminotransferase (U/L) | 27 (16, 36) | 20 (12, 41) | 0.3 |
| Total bilirubin (mg/dL) | 14 (9, 24) | 15 (10, 23) | 0.8 |
| Total protein(g/L) | 60 (52, 65) | 62 (54, 66) | 0.4 |
| Albumin(g/L) | 31.4 (28.3, 35.9) | 33.1 (29.2, 36.2) | 0.3 |
| C-reactive protein(mg/L) | 58 (28, 90) | 56 (20, 90) | 0.3 |
| Procalcitonin(ng/mL) | 0.55 (0.25, 2.78) | 0.76 (0.23, 2.19) | 0.9 |
| **Outcomes** |  |  |  |
| All-cause in-hospital mortality | 22 (36.1%) | 12 (19.7%) | 0.13 |
| 28-day mortality | 20 (32.8%) | 7 (11.5%) | 0.0012 |
| 14-day mortality | 18 (29.5) | 6 (9.8%) | 0.0014 |
| ^1^Data are median (IQR) for continuous variables and number of patients(%) for categorical variables. | | | |
| ^2^The data were analyzed using Wilcoxon rank sum test, Pearson's Chi-squared test and Fisher's exact test, respectively. | | | |

|  | | |  |  | |  | |
| --- | --- | --- | --- | --- | --- | --- | --- |
| Table S3 Primer sequences of adeN gene for PCR reaction | | | | | | | |
|  |  |  | | |  | |  |
| Primer Name | Sequence（5’→3’） | Annealing temperature（℃） | | | Amplicon size (bp) | | Reference |
| adeN-IS | F: ATGCATGATCCAGTCCTTGA | 50 | | | 654 to 1843 | | This study |
|  | R: TTAGACTTTATGATGCCCCT |  |  |  |  |  |  |
